# Control of Photoluminescence of Carbon Nanodots via Surface Functionalization using Para-substituted Anilines

**DOI:** 10.1038/srep12604

**Published:** 2015-07-28

**Authors:** Woosung Kwon, Sungan Do, Ji-Hee Kim, Mun Seok Jeong, Shi-Woo Rhee

**Affiliations:** 1 Department of Chemical Engineering, Pohang University of Science and Technology (POSTECH), 77 Cheongam-ro, Nam-gu, Pohang 790-784, South Korea; 2 Center for Integrated Nanostructure Physics, Institute for Basic Science (IBS), 2066 Seobu-ro, Jangan-gu, Sungkyunkwan University, Suwon 440-746, South Korea; 3 Department of Energy Science, Sungkyunkwan University, 2066 Seobu-ro, Jangan-gu, Suwon 440-746, South Korea

## Abstract

Carbon nanodots (C-dots) are a kind of fluorescent carbon nanomaterials, composed of polyaromatic carbon domains surrounded by amorphous carbon frames, and have attracted a great deal of attention because of their interesting properties. There are still, however, challenges ahead such as blue-biased photoluminescence, spectral broadness, undefined energy gaps and etc. In this report, we chemically modify the surface of C-dots with a series of *para*-substituted anilines to control their photoluminescence. Our surface functionalization endows our C-dots with new energy levels, exhibiting long-wavelength (up to 650 nm) photoluminescence of very narrow spectral widths. The roles of *para*-substituted anilines and their substituents in developing such energy levels are thoroughly studied by using transient absorption spectroscopy. We finally demonstrate light-emitting devices exploiting our C-dots as a phosphor, converting UV light to a variety of colors with internal quantum yields of ca. 20%.

Carbon nanodots (C-dots) are a new class of fluorescent carbon nanomaterials, attracting tremendous attention because of their excellent biocompatibility, innocuousness and photostability^1^,^2^. They have been recently described as paracrystalline carbon, composed of angstrom-sized polyaromatic carbon domains surrounded by amorphous carbon frames; however, their precise structure is still debatable^3^,^4^. While general paracrystalline carbons (e.g., coal, carbon black and etc.) are hardly fluorescent, their nanometer-sized debris have exhibited a certain “optical” energy gap, presumably depending upon their size, shape and surface states, due to either the quantum confinement or the auxochromic effect^5^,^6^,^7^,^8^,^9^. This artificial band gap leads to a number of interesting properties such as strong UV absorption, blue-to-green photoluminescence, excitation-energy-dependent photoluminescence and so on^10^,^11^,^12^,^13^,^14^. As a consequence, C-dots have shown strong potential for bioimaging, photovoltaics and optoelectronics and there are now tens of related applications in primary trials^15^,^16^,^17^,^18^,^19^,^20^,^21^.

Since Scrivens and coworkers first reported the photoluminescence of arc-discharged soot purified by electrophoresis^22^, there have been a number of synthetic methods for preparing C-dots, including laser ablation^23^, electrochemical oxidation^24^,^25^, solvothermal carbonization^26^,^27^,^28^, wet oxidation^5^,^7^,^29^,^30^, supported or templated synthesis^31^,^32^,^33^, microwave synthesis^34^,^35^,^36^ and so on. These methods have generally offered highly fluorescent and uniformly dispersed C-dots, but any of them still has no comparative advantage in terms of “coloration”. Here, coloration is defined as the diversity in colors of photoluminescence and it is limited up to green or yellow^2^. Although few groups have recently reported meaningful shifts in photoluminescence, there are still challenges ahead such as spectral broadness, undefined energy gaps and mechanistic ambiguity^37^,^38^,^39^,^40^,^41^,^42^.

The color of photoluminescence is directly related to the energy gap between two different energy levels. It has been reported that the energy gap of C-dots may primarily originate from their surface states^6^. Although the term “surface state” itself is unambiguous (defined as electronic states found at the surface), C-dots possess a variety of surface functional groups, defects and sometimes even adsorbates and hence, their surface states can hardly be identified in terms of physical and chemical properties. Such complexity is the main reason for the fact that our understanding of C-dots, especially regarding the role of the surface states in their photoluminescence, is still incomplete. Furthermore, the surface states of C-dots are mostly restricted within hydrophilic functional groups (e.g., carboxylic acid, aldehyde, alcohol and etc.) because almost all synthetic methods that have been widely used employ water as a reaction medium^43^. As a consequence, molecules that could be used in surface functionalization have been limited to being water soluble.

If C-dots are to be considered as potential fluorescent materials for imaging and optoelectronics, there is an urgent need to improve their coloration. We hypothesized that the introduction of different surface functional groups would lead to electronic changes in the energy gap and, as a result, the coloration might be extended over a wider range of the visible spectrum. We now report that the photoluminescence of C-dots can be chemically controlled by the introduction of a series of *para*-substituted anilines onto the surface. Our surface functionalization would give rise to substantial changes in the photoluminescence, showing yellow-to-red photoluminescence of very narrow spectral widths. Our results indicated that surface functional groups play a very major role in the photoluminescence of C-dots, providing important information about its origin.

## Results

**Figure 1:**
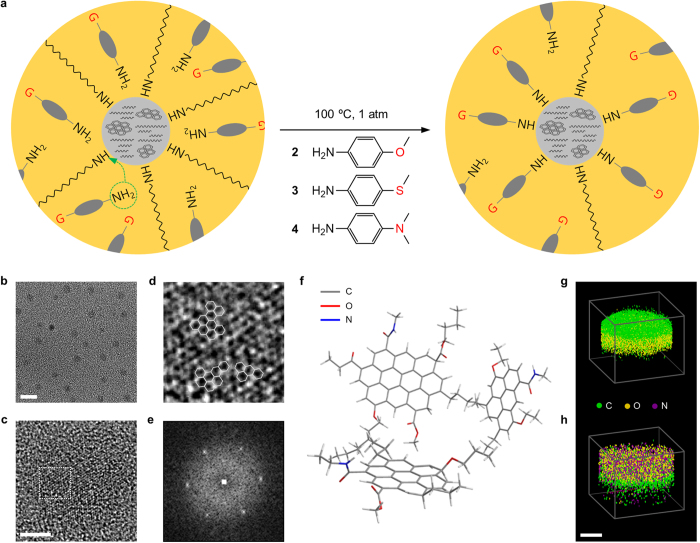
Synthetic scheme and structure. (**a**) Schematic of surface functionalization and molecular structures of *para*-substituted anilines used in the experiments. (**b**–**d**) TEM images of C-dots (**b**) and their polyaromatic carbon domains (**c**,**d**). The scale bars in (**b**,**c**) represent 5 and 1 nm, respectively. The area surrounded by the dotted square in **c** is magnified in (**d**). The solid hexagons in (**d**) represent benzene. (**e**) Diffraction pattern of a polyaromatic carbon domain. (**f**) Chemical structure of C-dots. (**g**,**h**) Atom probe tomography of C-dots before (**g**) and after surface functionalization (**h**). The scale bar represents 5 nm.

In our approach, oleylamine-capped C-dots (**1**) were prepared by solvothermal carbonization of citric acid^33^. They were then subject to surface functionalization with a series of *para*-substituted anilines, as illustrated in Fig. 1a (see Methods for details). We here chose 4-methoxyaniline (**2**), 4-(methylthio)aniline (**3**) and 4-(dimethylamino)aniline (**4**) because of their same head group, i.e., we could isolate the effect of the substituent on the electronic and optical properties of C-dots. In Fig. 1b, the transmission electron microscopy (TEM) images show that no physical change was led in C-dots by surface functionalization (see Supplementary Fig. S1 for more images). Their size was ca. 2–3 nm (Supplementary Fig. S2) and structure showed weak sign of graphitic carbon due to our relatively low carbonization temperature (Fig. 1c). According to Oberlin, our C-dots might be composed of polyaromatic carbon domains of 6 to10 benzene rings and amorphous carbon networks^44^. Such polyaromatic carbon domains were found in reality (Fig. 1d), showing a typical diffraction pattern of graphitic carbon with short-range order (Fig. 1e). Raman spectroscopy also confirmed the paracrystalline nature of our C-dots (Supplementary Fig. S3). As a result, we could present the chemical structure of our C-dots, as illustrated in Fig. 1f.

Atom probe tomography visualized an atomic distribution in a volume of C-dots, indicating that the number of nitrogen atoms was grown after surface functionalization (Fig. 1g,h). This could be attributed to the fact that the substituted anilines experienced much less steric hindrance than oleylamine during surface functionalization. The x-ray photoelectron spectroscopic data consistently showed that the atomic ratio of nitrogen was increased by 4–10% after surface functionalization (Supplementary Fig. S4 and Table S1), while the signs of carbon-carbon and carbon-oxygen bondings were preserved (Supplementary Fig. S5), indicating that there was no significant change in the chemical structure of the core. The nitrogen (1s) signals show a peak at around 401 eV, presumably assigned to acyl nitrogen (N−C=O), indicating that the amine groups of oleylamine and the substituted anilines were bonded with carbonyl groups (from citric acid) to form amide groups on the surface of C-dots (Supplementary Fig. S4). The deconvoluted carbon (1s) spectra also indicated that C−C and C=C (284 eV), C=O and C−O (288 eV) and C−N (286 eV) bondings were present on the surface of C-dots (Supplementary Fig. S5). We sought evidence for the chemical bonding of the substituted anilines on the surface of C-dots from carbon-13 nuclear magnetic resonance data. In Supplementary Fig. S6, **1** showed chemical shifts of 14.1, 22.7, 31.9, 32.6 (alkyl) and 130.4 ppm (alkene) for the oleyl group and 38.9, 39.1 and 42.8 ppm for amido carbon, indicating that oleylamine was bonded to carboxylic acid on the surface of C-dots^33^. After surface functionalization, **2**, **3** and **4** had lines at around 14.1, 55.6 and 41.3 ppm for the methoxy, methylthio and methylamino groups, respectively, while they still showed the oleyl and amido shifts (Supplementary Fig. S7). This implies that the substituted anilines would rather be bonded to vacancies than replace oleylamine because such amide bonds can be hardly broken under our experimental condition. Infrared spectroscopy might also provide a clue to the understanding of the chemistry of surface functionalization. In Supplementary Fig. S8, **2**, **3** and **4** showed aromatic C−C and C−N stretching bands of aniline at around 1500−1400 and 1350−1250 cm^−1^, respectively, while they preserved the trace of the C−H stretching band of the oleyl group at around 3000–2900 cm^−1^.

In organic molecules, their energy gaps are regarded as electronic transitions from one energy level to a higher energy level. Such electronic transitions involve bonding (σ and π), antibonding (σ* and π*) and nonbonding (*n*) orbitals, where σ → σ*, π → π*, *n* → σ* and *n* → π* transitions generally take place. Among them, π → π* and *n* → π* transitions are of particular concern because their transition energies correspond to the wavelengths of visible light, i.e., germane to photoluminescence in the range of the visible spectrum. In reality, all such transitions are correlated with each other to generate the ground (S_00_), first excited (S_11_), second excited (S_22_) states and so forth.

**Figure 2:**
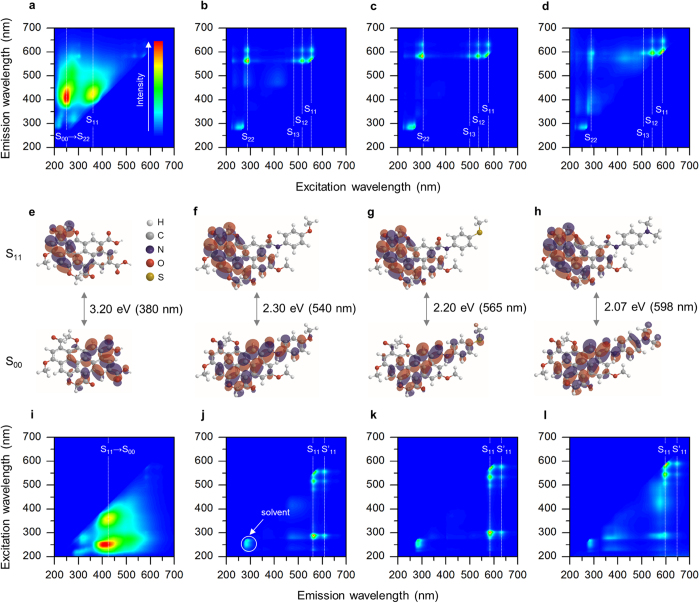
Photoluminescence spectroscopy analyses. (**a**–**h**) Excitation maps (**a**–**d**) and their corresponding energy level diagrams (**e**–**h**) of **1** (**a**,**e**), **2** (**b**,**f**), **3** (**c**,**g**) and **4** (**d**,**h**). The ground, first excited and second excited states are denoted as S_00_, S_11_ and S_22_, respectively. The dotted lines guide the eye. (**i**–**l**) Emission maps of **1** (**i**), **2** (**j**), **3** (**k**) and **4** (**l**).

**Figure 3:**
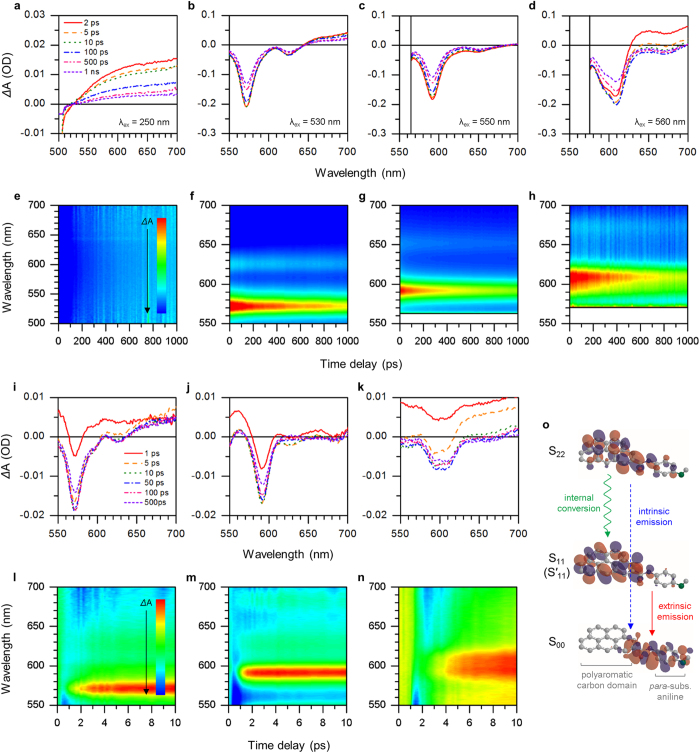
TA spectroscopy analyses. (**a**–**d**) TA spectra at different excitation wavelengths (λ_ex_) and (**e**–**h**) corresponding time-correlated excited-state electron density maps of **1** (**a**,**e**), **2** (**b**,**f**), **3** (**c**,**g**) and **4** (**d**,**h**). (**i**–**k**) TA spectra at the excitation wavelength of 300 nm and (**l**–**n**) corresponding time-correlated excited-state electron density maps of **2** (**i**,**l**), **3** (**j**,**m**) and **4** (**k**,**n**). Red color represents high electron density (negative *Δ*A), while blue color represents the opposite (positive *Δ*A). (**o**) Energy level diagram of surface functionalized C-dots. The molecular orbitals are assumed.

**Figure 4:**
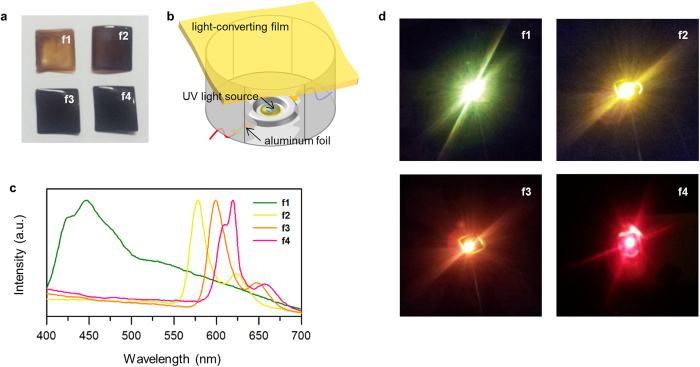
LED demonstrations. (**a**) Photos of light-converting films. (**b**) Construction of LEDs. (**c**) Emission spectra of LEDs. The intensity is normalized for easy comparison. (**d**) Photos of LEDs during operation.

To explore the electronic transitions in our C-dots, we undertook a series of photoluminescence spectroscopy measurements (Fig. 2 and Supplementary Fig. S9). In Fig. 2a, the excitation map (a set of excitation spectra) of **1** shows two bands at the excitation wavelengths of 250 and 360 nm, corresponding to the photon energies of 4.96 and 3.44 eV, respectively. Recalling that our C-dots possess 6 to 10 fused benzene rings of which π−π* gap has been known to be ca. 5.0 eV^45^, the first band (S_00_ → S_22_) could represent the π → π* transition. The low-energy S_11_ transition might involve *n* orbitals, where our C-dots contained a significant amount of oxygen and nitrogen atoms. These atoms could also form a variety of chemical structures with different energy levels, responsible for the broadness of the bands. Fig. 2b–d show that surface functionalization would lead very dramatic changes to the electronic transition. The broad bands of **1** were dimmed and instead very sharp, structured peaks were observed, which implies that the substituted anilines almost entirely “covered” the surface of C-dots and they behaved as a well-defined dye molecule. These new peaks might also be categorized into π → π* (S_22_) and *n* → π* (S_11–13_, including vibrational modes) transitions by means of the light absorption spectra (Supplementary Fig. S10). We noted that after surface functionalization, S_22_ was red-shifted by 38–50 nm, presumably due to the bathochromic effect of the electron donating groups of the substituted anilines. The degree of such bathochromic shift was inversely proportional to the electronegativity of the substituents, where S_22_ of **3** (−SCH_3_, Fig. 2c) was more red-shifted than that of **2** (−OCH_3_, Fig. 2b) and **4** (−N(CH_3_)_2_, Fig. 2d)^46^. It is worth noting that S_11–13_ were also a function of the substituents, e.g. S_11_ of **2**, **3** and **4** was detected at 555, 575 and 590 nm, respectively. In Fig. 3e–h, the Hückel calculation nicely simulated the change in the S_00_–S_11_ gap upon the substituents (Table S2), implying that their chemical properties (atomic radius, electronegativity, ionization energy and etc.) had a significant effect on the transition energy required to redistribute molecular orbitals.

In Fig. 2i, the emission map (a set of emission spectra) of **1** shows two broad bands at the same emission wavelength of 420 nm (S_11_ → S_00_) regardless of the excitation wavelength, indicating that Kasha’s rule was met. Fig. 2j–l show that after surface functionalization, such “intrinsic” emission disappeared while very narrow “extrinsic” emission was observed at longer wavelengths. The intensity ratio of extrinsic to intrinsic emission was increased with the degree of surface functionalization (Supplementary Fig. S11) that could be determined by nuclear magnetic resonance and x-ray photoelectron spectroscopy analyses (Supplementary Fig. S12 and Table S3). This proved that there were at least two photoluminescent centers interacting with each other competitively^47^. In other words, it was concluded that intrinsic emission was attributed to “intrinsic” surface functional groups (and its broadness was due to their chemical variety), but could be refined by homogenizing the surface of C-dots to result in extrinsic emission. Such extrinsic emission was composed of two vibrational bands, where the major peaks (S_11_) of **2**, **3** and **4** were observed at 565 (Fig. 2j), 590 (Fig. 2k) and 600 nm (Fig. 2l), respectively. The minor peaks (S′_11_) were detected at longer (ca. 50 nm) wavelengths with the corresponding energy difference of 0.1–0.2 eV, analogous to the C−C inter-ring or intra-ring stretch modes of polyfluorene^48^,^49^. We noted again that the peak wavelengths were determined by the substituents, where **4** showed the most reddish photoluminescence. This can be attributed to the fact that its substituent (−N(CH_3_)_2_) is the strongest π donor and so the most capable of reducing the energy gap bathochromically. Similarly, **3** was more reddish than **2** because sulfur could be a stronger π donor than oxygen if adjacent to (partially) positive carbon^50^. The real photo of C-dots under 360 nm UV irradiation was presented in Supplementary Fig. S13. The quantum yields of C-dots were slightly decreased after surface functionalization (ca. 40% for **1**) because there was a loss of photoexcited electrons during internal conversion^33^. Interestingly, **3** recorded a higher quantum yield (33%) than **2** (31%) and **4** (21%) because sulfur has lower electronegativity and its electrons can be more readily excited by photons (Supplementary Fig. S14). Consistently, halogen substituents (F, Cl and Br) induced blue-shift in photoluminescence and lowered its intensity because they are weakly deactivating and their electrons are hardly accessible (Supplementary Fig. S15).

To further explore the origin of extrinsic emission, we conducted transient absorption (TA) spectroscopy measurements (Fig. 3). For **1**, its *Δ*A values were recorded positive over 520 nm on all time scales (Fig. 3a), indicating that its photoexcited electrons were not allowed to access lower energy levels and just “intrinsically” consumed to emit blue-to-green (<520 nm) photoluminescence. In Fig. 3b, **2** exhibited two strong negative peaks at around 570 and 620 nm, coinciding with its emission peaks (S_11_ and S′_11_), respectively. It may be seen clearly that **3** (Fig. 3c) and **4** (Fig. 3d) follow a similar trend, where their *Δ*A values slowly get close to zero over the observation period. This implied that these new, extrinsic energy levels formed by the substituted anilines were directly related with the long-wavelength photoluminescence. We calculated the decay time of photoexcited electrons in our C-dots by fitting the TA data (Supplementary Fig. S16 and Table S4). Each decay time in **2**, **3** and **4** was approximately three times longer than that in **1**, which supports our hypothesis that surface functionalization would decrease the number of defective states causing non-radiative dissipation through trapping, phonon scattering, or other feasible dissipation routes. It is worth noting that S’_11_ (solid circles) had shorter decay times than S_11_ (open circles). This proved that, if considering that S’_11_ had never appeared solely without S_11_, S’_11_ was a kind of vibrational states introduced by surface functionalization, or specifically, the substituted anilines. The time-correlated excited-state electron density maps were presented to visualize corresponding energy levels and their excited-state photodynamics (Fig. 3e–h). In addition, time-resolved photoluminescence (TRPL) spectroscopy measurements were conducted to reveal the dynamics of radiative recombination (Fig. S17 and Table S5). The TRPL spectra were well fitted to single exponential functions, indicating that the S_11_ → S_00_ (extrinsic to ground state) recombination occurred dominantly. The photoluminescence lifetimes of our C-dots were several (2–4) nanoseconds, few times longer than the decay times obtained by the TA analyses earlier. This verified that photoexcited electrons were relaxed into photoluminescent centers (here, S_11_) through non-radiative processes and settled for a while before their radiative recombination, responsible for the high quantum yields of our C-dots >20%. We also note that such photoluminescence decays are detected at the peak wavelengths of extrinsic emission, indicating that strong, negative *Δ*A of the TA spectra could be attributed to ground-state bleaching.

To study the correlation between intrinsic and extrinsic energy levels, we recorded TA spectra at a short excitation wavelength (300 nm) that could allow direct access to intrinsic energy levels. Fig. 3i–k show that the signals grew downward in the very first stage (1–10 ps), reached their peak at around 50 ps and then dropped off to zero after 100 ps. Consistently, the decay time of short-lived excitons (τ_1_) was significantly decreased, while that of long-lived excitons (τ_2_) was kept almost constant (Supplementary Fig. S18 and Table S6). These results might indicate that there was relaxation of such high-energy photoexcited electrons to low-energy extrinsic energy levels in few picoseconds (internal conversion) with a certain degree of dissipation. Such internal conversion and related time delay were likewise observed in Fig. 3l–n, which could support our hypothesis that two photoluminescent centers (intrinsic and extrinsic) co-existed and worked in a competitive manner. We finally presented the energy level diagram of our C-dots in Fig. 3o.

We demonstrated light-emitting devices (LEDs) exploiting our C-dots as a phosphor to convert UV to visible light with a variety of colors. To facilitate the handling of C-dots, they were embedded in poly(methyl methacrylate) matrices to form flexible, freestanding light-converting films (Fig. 4a). We denoted the films of **1**, **2**, **3** and **4** as **f1**, **f2**, **f3** and **f4**, respectively. Fig. 4b shows the construction of LEDs. The UV light sources (280–320 nm) were optimized to achieve maximum intensity, operating at the applied bias of 6 V (~50 mA). We found that the emission spectra of LEDs strongly reflected the photoluminescence of C-dots, but there were 5–30 nm red-shifts presumably due to the inner-filter effect (Fig. 4c). As a result, our LEDs with **f1**, **f2**, **f3** and **f4** successfully exhibited green, yellow, orange and red light, respectively (Fig. 4d), of which corresponding CIE (Commission Internationale de l’Éclairage) chromaticity coordinates were presented in Supplementary Fig. S19. The internal quantum yields of our films were recorded 12–26% (Supplementary Fig. S20), comparable to those of conventional phosphors often containing toxic or expensive metals such as europium, cadmium, lead, terbium and so on. Furthermore, our films showed outstanding stability against UV light, air, water and heat, which would realize long-term operation for >24 h in practical conditions without losing their properties (Supplementary Fig. S21).

## Discussion

Our results showed that the *para*-substituted anilines could cause a dramatic change in the energy levels of C-dots and their *para*-substituents seemed to determine the wavelength of photoluminescence. To verify the role of substituents, we introduced aniline (no substituent) molecules onto the surface of our C-dots. In Supplementary Fig. S22, the emission map showed no sign of any extrinsic emission, implying that *n* orbitals of substituents played a central role in such long-wavelength photoluminescence. If we hypothesize that *n* orbitals are conjugated with polyaromatic carbon domains, the wavelength of extrinsic emission should be a function of the activating ability of the outermost functional groups (e.g., methyl for **2**, **3** and **4**). To confirm our hypothesis, 4-(octyloxy)aniline was employed, of which outermost octyl is inductively more electron donating than methyl. Supplementary Fig. S23 shows that 4-(octyloxy)aniline resulted in a sensible red-shift (ca. 10 nm) compared to 4-methoxyaniline, proving that *n* orbitals and their lone pairs of electrons were donated to the core of C-dots to constitute extrinsic energy levels. The importance of conjugation between substituents and polyaromatic carbon domains could be confirmed by introducing benzylamine molecules, of which additional CH_2_ groups were expected to deliberately intervene the delocalization of electrons over C-dots. In this regard, we employed 4-methoxybenzylamine and found that there was just intrinsic emission similar to **1** (Supplementary Fig. S24). This result may strongly support our hypothesis that the *para*-substituted anilines, specifically their substituents, were coupled with intrinsic energy levels *via* conjugation to result in extrinsic emission eventually. Thus, introducing more substituted aniline molecules onto the surface of C-dots could constitute more extrinsic energy levels “with” sacrificing intrinsic energy levels and so, the intensity ratio of extrinsic to intrinsic emission was a function of the degree of surface functionalization (Supplementary Fig. S11).

The formation of extrinsic energy levels was further confirmed by electron energy loss spectroscopy. Supplementary Fig. S25 shows that there was a significant increase in the number of electrons with binding energies of 5–6 eV after surface functionalization. The energy gaps were roughly calculated to be 2.0–2.5 eV, consistent with the wavelengths of extrinsic emission (560–600 nm). Ultraviolet photoelectron spectroscopy also indicated that the energy levels of valence electrons of C-dots were around 6 eV, and shifted upward after surface functionalization (Supplementary Fig. S26). The Kelvin probe analyses indicated that the Fermi levels of our C-dots were 4.70–4.75 eV (Supplementary Fig. S27), confirming their energy levels and gaps that we proposed based on our spectroscopy measurements where Ishii and co-workers have reported that the bulk Fermi level of any organic semiconductor could be assumed to be placed at the midst of its S_00_–S_11_ gap at room temperature^51^.

In summary, we have improved the coloration of C-dots via surface functionalization employing a series of *para*-substituted anilines. The photoluminescence of C-dots was dramatically changed by surface functionalization not only in color (blue to warm colors) but also in spectral width (broad to narrow). It was clearly seen by TA spectroscopy that such changes could be attributed to the formation of new energy levels that were denoted as extrinsic due to their origin. These extrinsic energy levels relied on *para*-substituents and interacted with original, intrinsic energy levels in a competitive manner, where we detected internal conversion of photoexcited electrons from intrinsic to extrinsic energy levels. We finally demonstrated light-emitting devices exploiting our C-dots as a phosphor to efficiently convert UV light to a variety of colors with the aid of their excellent fluorescent quantum yield and long-term stability. Our results might pave the way for understanding the origin of the photoluminescence of C-dots and extending the boundary of their practical use in a variety of areas such as bioimaging, displaying, lighting and so forth.

## Methods

### Synthesis

A three-neck round-bottom flask was charged with citric acid (1 g) and water (1 ml). Once citric acid was dissolved completely, 0.5 M nitric acid (1 ml), oleylamine (1 ml) and 1-octadecene (9 ml) were added. The mixture was allowed to stir for 30 min and heated for 3 h at 250 °C. After cooling to room temperature, the mixture was transferred into conical tubes, thoroughly mixed with methanol (~80 ml) and then centrifuged at 3,000 rpm for 15 min to remove excess reagents. This purification process was repeated three times. The mixture was dried for 12 h at 80 °C under vacuum to yield **1**.

### Surface functionalization

**1** (15 mg) was dissolved in toluene (3 ml). To the solution was added 4-methoxyaniline (2.14 g, 15 mmol). The solution was heated for 12 h at 100 °C under vigorous stirring. The temperature was monitored by a thermocouple digital temperature probe. After cooling to room temperature, the remaining solid was dissolved in toluene (3 ml) and then dialyzed against toluene for at least 72 h by using Spectra/Por Biotech Cellulose Ester dialysis tubes (100–500 Da). The solution was evaporated to dryness on a rotary evaporator to yield **2**. 4-(methylthio)aniline (2.24 g, 15 mmol) and 4-(dimethylamino)aniline (2.18 g, 15 mmol) were used, instead of 4-methoxyaniline, for **3** and **4**, respectively, while all other experimental conditions were unchanged.

### Atom probe tomography

C-dots (10 mg) were dissolved in hexane (1 ml). Silicon substrates were spin-coated with a C-dot film (50 nm) from the solution at 1,000 rpm. The spin-coated substrates were then coated with aluminum (120 nm) by thermal evaporation. The substrates were shaped into a needle (20 nm in diameter) by dual-beam focused ion beam milling (Helios Nanolab 650) to give atom probe tomography specimens (Supplementary Fig. S27). Atom probe tomography was performed by using Cameca LA-WATAP.

### Characterizations

TEM was performed by using Jeol JEM-2200FS equipped with a Cs corrector. Raman spectroscopy was performed by using a Witec Alpha 300R spectrometer with a laser excitation wavelength of 785 nm. X-ray photoelectron spectroscopy was performed by using an Escalab 250 spectrometer with an Al x-ray source (1486.6 eV). Nuclear magnetic resonance spectra were recorded on a Bruker DRX500 spectrometer (500 MHz). Infrared spectroscopy was performed by using a Nicolet 6700 FT-IR spectrometer equipped with a demountable cell (Part Num. 162-3600) with a pair of KBr windows (Pike Technologies). Kelvin probe force microscopy was performed by using an SKP5050 Scanning Calvin probe (KP Technology). Electron energy loss and ultraviolet photoelectron spectroscopies were carried out in an ultra-high-vacuum chamber equipped with a VUV-5000 generator (40.8 eV He II laser) and a SES-100 detector. Detailed procedures for sample preparation were presented in Supplementary Methods.

### Light absorption and photoluminescence spectroscopy

10 mm × 10 mm QS-grade quartz cuvettes (Hellma Analytics 111-QS) were used. Hexane was used as a solvent. Light absorption spectra were recorded on a Scinco S-3100 spectrophotometer. Photoluminescence spectra were recorded on a Jasco FP-8500 fluorometer.

### Transient absorption spectroscopy

Transient absorption spectroscopy was measured by using a visible Helios system (Ultrafast systems) with a 1 kHz femtosecond Ti:sapphire laser system (Libra), having 780 nm center wavelength and 80 fs pulse duration. The laser beam was divided by a beam splitter. About 95% of the laser beam drove an optical parametric amplifier (TOPAS Prime) to be used as a tunable pump beam (250–560 nm) with the pulse duration of 200 fs. The other 5% was focused on a 5-mm sapphire crystal to generate a white-light continuum to be used as a probe beam (400–750 nm).

### Time-resolved photoluminescence spectroscopy

Time-resolved photoluminescence spectroscopy was measured by using the same laser setup above. The data were recorded on a Princeton Instruments SP2300 spectrometer combined with a Hamamatsu C5680 streak camera.

### Quantum yield measurements

2 mm × 10 mm QS-grade quartz cuvettes (Jasco Parts Center 6808-H250A) were used. Absolute quantum yields were recorded on a Jasco FP-8500 fluorometer equipped with a 100 mm integrating sphere setup (ILF-835) and calculated by using Jasco Spectra Manager II Software.

### Fabrication and characterization of light-emitting devices

C-dots (250 mg) were dissolved in toluene (5 ml). The solution (5 ml) was mixed with the 10% poly(methyl methacrylate) solution in anisole (5 ml). The mixture was vortexed for 10 min and air bubbles were removed. The mixture (0.5 ml) was then dropped onto 18 mm × 18 mm cover glasses and kept undisturbed for 24 h on a flat table. Once the solvent was evaporated completely, the resulting films were peeled off from the cover glasses and placed over 280–320 nm InGaN light-emitting diodes by using adhesive tapes. Emission spectra were recorded on a Minolta CS2000 spectroradiometer with a Keithley 236 sourcemeter. Internal quantum yields were recorded on a Jasco FP-8500 fluorometer equipped with a 100 mm integrating sphere setup (ILF-835) and calculated by using Jasco Spectra Manager II Software.

## Additional Information

**How to cite this article**: Kwon, W. *et al.* Control of Photoluminescence of Carbon Nanodots via Surface Functionalization using Para-substituted Anilines. *Sci. Rep.* **5**, 12604; doi: 10.1038/srep12604 (2015).

## Supplementary Material

Supplementary Information
