# Supplementary material for: Control of Photoluminescence of Carbon Nanodots via Surface Functionalization using Para-substituted Anilines
Source: Sci Rep. 2015 Jul 28;5:12604. doi: 10.1038/srep12604 (PMC4517466; doi:10.1038/srep12604)
Supplement: Supplementary Information [file srep12604-s1.pdf]

Supplementary Information for

Control of Photoluminescence of Carbon Nanodots via Surface Functionalization using Para-substituted Anilines

Woosung Kwon[1], Sungan Do[1], Ji-Hee Kim[2], Mun Seok Jeong[2,3,★] and Shi-Woo Rhee[1,★]

[1]Department of Chemical Engineering, Pohang University of Science and Technology (POSTECH), 77 Cheongam-ro, Nam-gu, Pohang 790-784, South Korea.

[2]Center for Integrated Nanostructure Physics, Institute for Basic Science (IBS), 2066 Seobu-ro, Jangan-gu, Sungkyunkwan University, Suwon 440-746, South Korea.

[3]Department of Energy Science, Sungkyunkwan University, 2066 Seobu-ro, Jangan-gu, Suwon 440-746, South Korea.

Corresponding author footnote

[★]Corresponding author.

Tel: +82-31-299-4053; Fax: +82-31-299-6505; E-mail: mjeong@skku.edu (M.S.J.)

Tel: +82-54-279-2265; Fax: +82-54-279-8619; E-mail: srhee@postech.ac.kr (S-.W.R.)

Contents

Methods for sample preparation S1

Figure S1 .... S3

Figure S2 .... S4

Figure S3 .... S5

Figure S4 .... S6

Figure S5 .... S7

Figure S6 .... S8

Figure S7 .... S9

Figure S8 .... S10

Figure S9 .... S11

Figure S10 .... S12

Figure S11 .... S14

Figure S12 .... S16

Figure S13 .... S19

Figure S14 .... S20

Figure S15 .... S21

Figure S16 .... S22

Figure S17 .... S23

Figure S18 .... S24

Figure S19 .... S29

Figure S20 .... S30

Figure S21 .... S31

Figure S22 .... S32

Figure S23 .... S33

Figure S24 .... S34

Figure S25 .... S35

Figure S26 .... S36

Figure S27 .... S37

Figure S28 .... S38

Table S1 .... S6

Table S2 .... S13

Table S3 .... S18

Table S4 .... S22

Table S5 .... S23

Table S6 .... S24

Methods for sample preparation

Materials. All reagents were purchased from commercial sources and used without further purification. Citric acid (99.5%), 1-octadecene (95%), toluene (anhydrous, 99.5%) and 4-methoxyaniline (99%) were purchased from Sigma-Aldrich. Oleylamine (90%) was purchased from Acros Organics. 4-(methylthio)aniline (98%) was purchased from TCI. 4-(dimethylamino)aniline (96%) was purchased from Alfa Aesar. Poly(methyl methacrylate) (10% in anisole; molecular weight = 950,000) was purchased from Microchem. Deuterated chloroform (99.96%) was purchased from Cambridge Isotope Laboratories. Silicon wafers (150 mm in diameter) were purchased from Siltron Inc. and cut into 10 mm × 10 mm pieces by means of a diamond cutter. Gold wires (99.99% purity) were purchased from Koralco. A gold film (50 nm) was deposited onto silicon wafers by using thermal evaporator to give gold-coated silicon substrates. Water refers to deionized water.

TEM. C-dots (1 mg) were dissolved in hexane (1 ml). The solution (10 μl) was dropped onto a CF300-Cu TEM grid (Electron Microscopy Sciences) and dried for 12 h at 80 ℃ under vacuum.

Raman spectroscopy. C-dots (10 mg) were dissolved in toluene (1 ml). An aliquot of the solution (100 μl) was spin-cast on gold-coated silicon substrates and dried for 12 h under ambient conditions.

X-ray photoelectron spectroscopy. C-dots (10 mg) were dissolved in toluene (1 ml). An aliquot of the solution (100 μl) was spin-cast on gold-coated silicon substrates and dried for 12 h under ambient conditions.

Nuclear magnetic resonance. C-dots (100 mg) were dissolved in deuterated chloroform (1 ml). The solution was transferred into 600 MHz nuclear magnetic resonance sample tubes (Optima) and sealed by Teflon tapes.

Infrared spectroscopy. C-dots (100 mg) were dissolved in anhydrous toluene (1 ml). An aliquot of the solution (100 μl) was dropped onto a KBr window (Pike Technologies), dried for 12 h under high-purity nitrogen flow and sandwiched with another KBr window. This pair of KBr windows with C-dots was mounted on a demountable cell.

Kelvin probe force microscopy. C-dots (10 mg) were dissolved in toluene (1 ml). An aliquot of the solution (100 μl) was spin-cast on gold-coated silicon substrates and dried for 12 h under ambient conditions. The probe was calibrated by using a gold substrate.

Electron energy loss spectroscopy. C-dots (10 mg) were dissolved in toluene (1 ml). An aliquot of the solution (100 μl) was spin-cast on gold-coated silicon substrates and dried for 12 h under ambient conditions.

Ultraviolet photoelectron spectroscopy. C-dots (10 mg) were dissolved in toluene (1 ml). An aliquot of the solution (100 μl) was spin-cast on gold-coated silicon substrates and dried for 12 h under ambient conditions.

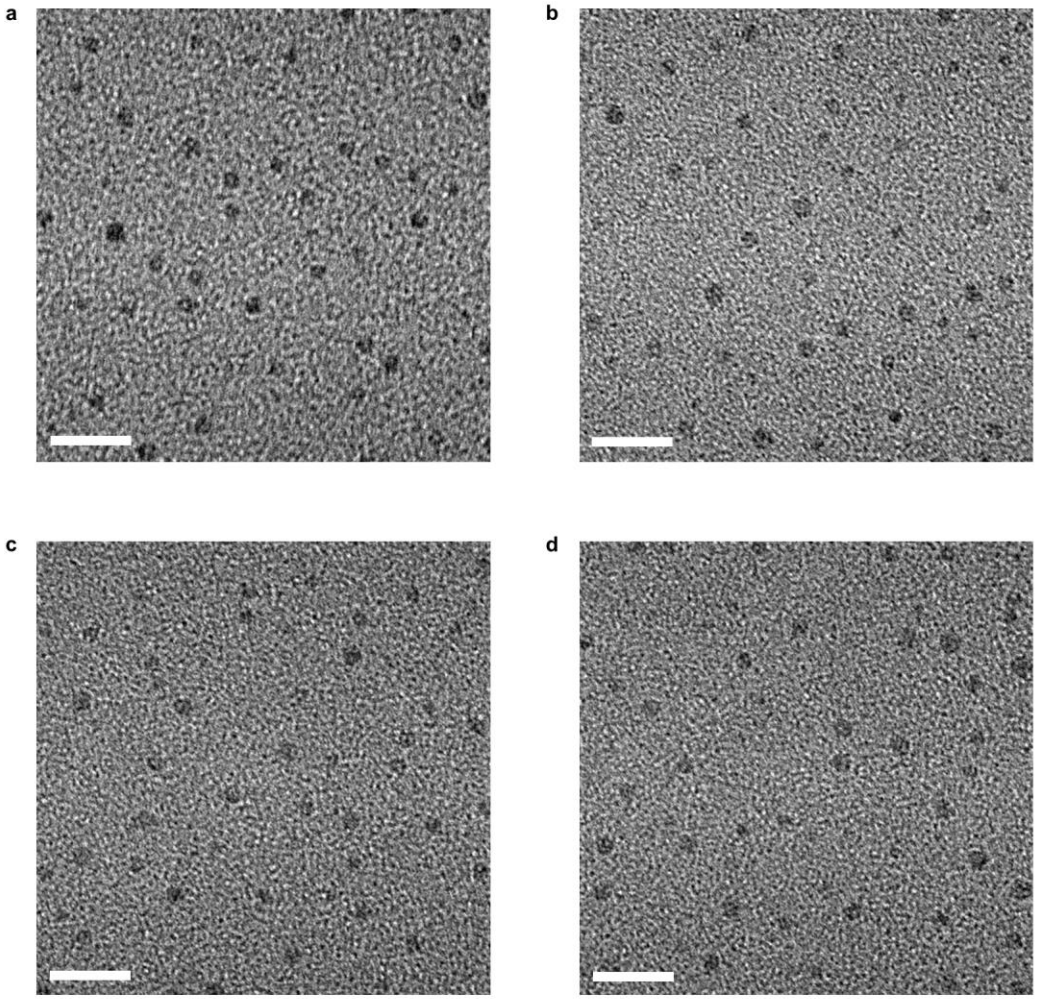

Figure S1. TEM images of **1** (**a**), **2** (**b**), **3** (**c**) and **4** (**d**). Scale bars, 10 nm.

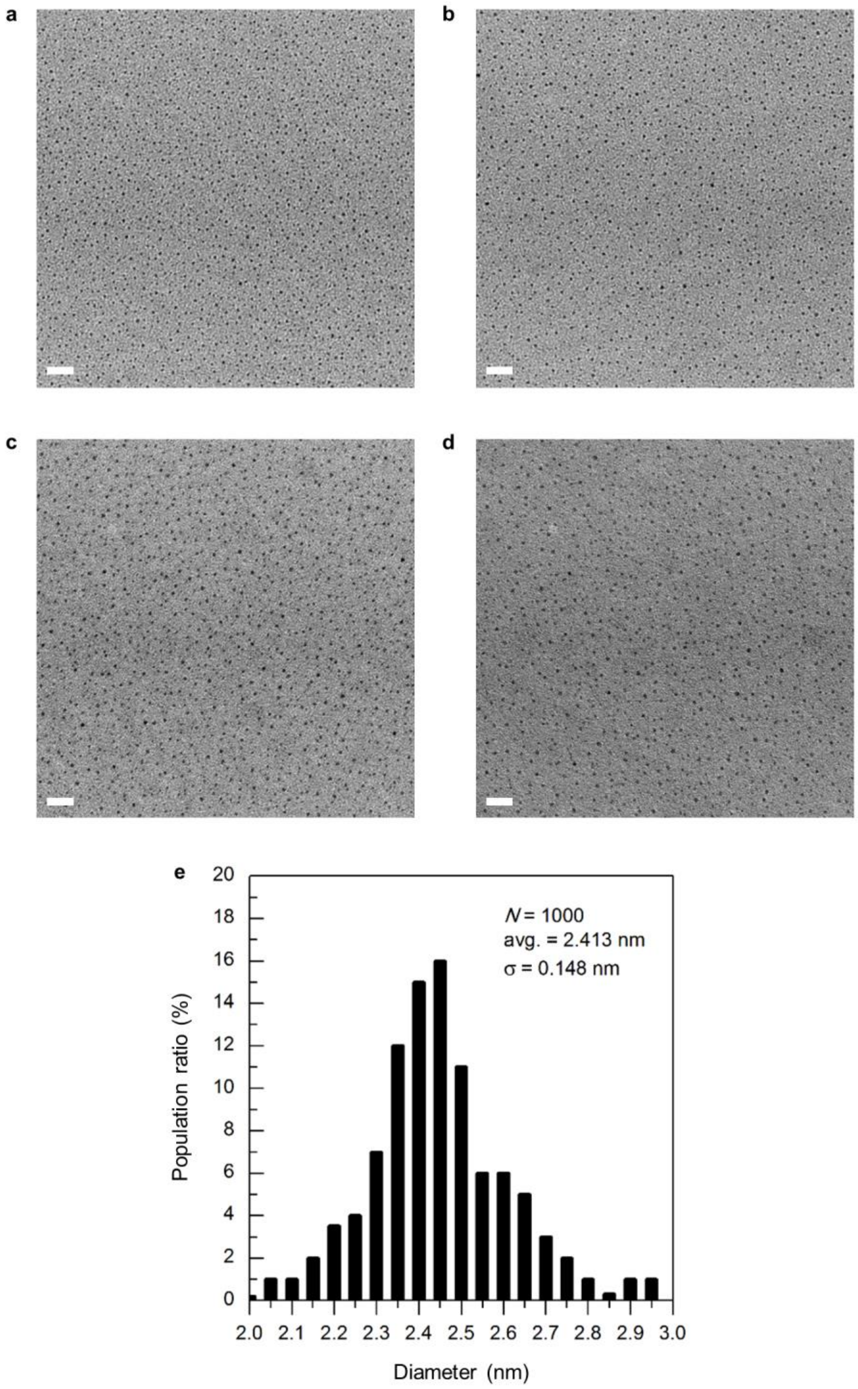

Figure S2. Low resolution TEM images of **1** (**a**), **2** (**b**), **3** (**c**) and **4** (**d**) for size statistics. Scale bars, 20 nm. (**e**) Size distribution of all samples. Population ratios below 0.5% are not shown.

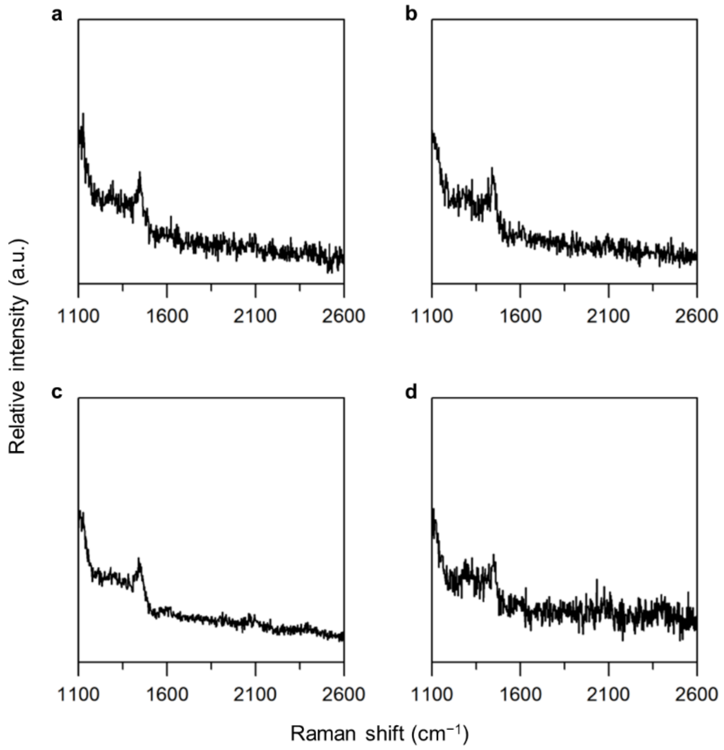

Figure S3. Raman spectra of **1** (**a**), **2** (**b**), **3** (**c**) and **4** (**d**).

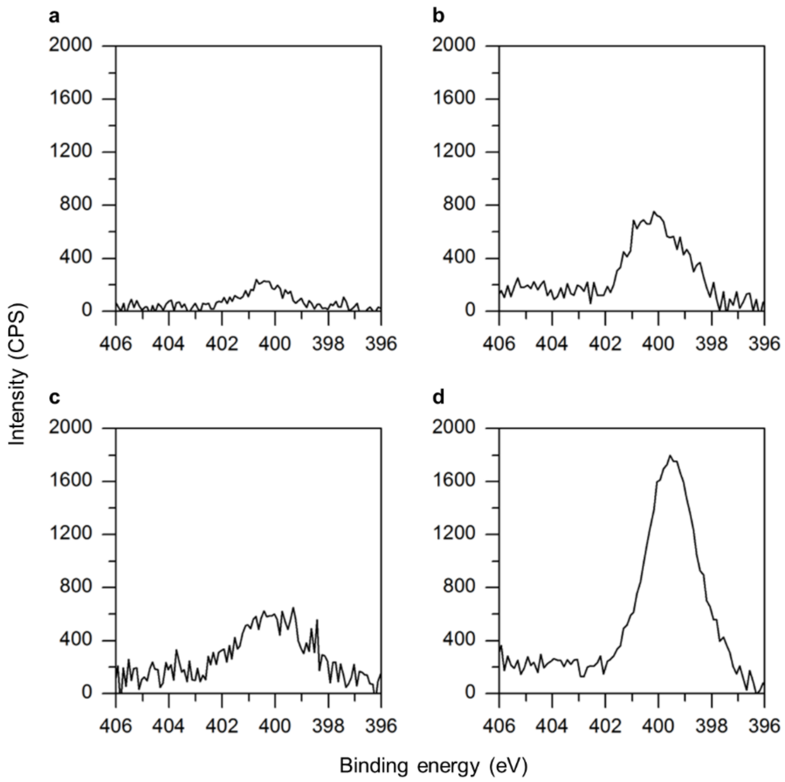

Figure S4. X-ray photoelectron spectra (N1s) of **1** (**a**), **2** (**b**), **3** (**c**) and **4** (**d**).

Table S1. Atomic ratios of C-dots.

Sample	Atomic ratio (%)			
	C1s	N1s	O1s	S2p
**1**	92	1.43	6.44	~0
**2**	77.91	6.82	14.30	~0
**3**	82.69	6.74	9.06	1.37
**4**	73.42	12.92	11.91	~0

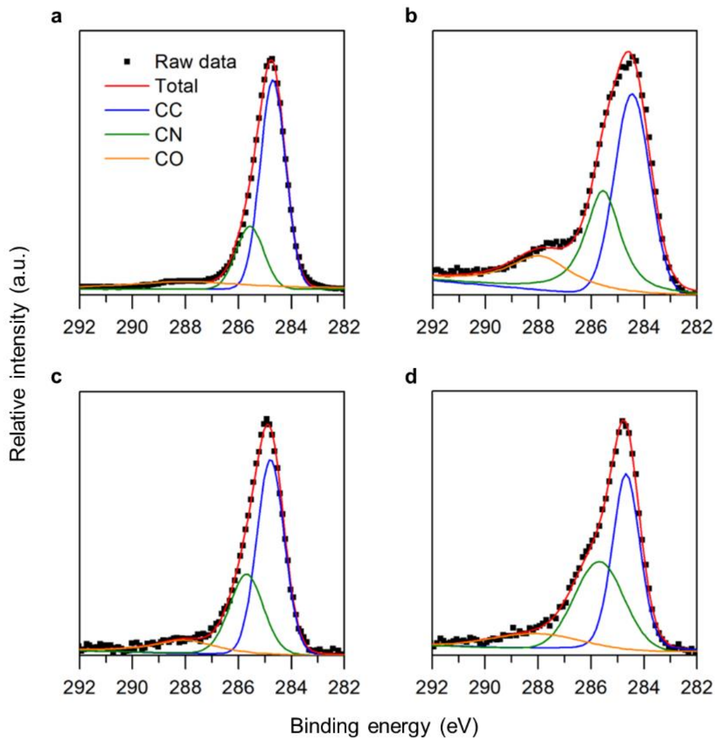

Figure S5. X-ray photoelectron spectra (C1s) of **1** (**a**), **2** (**b**), **3** (**c**) and **4** (**d**). Color coding represents chemical bondings and is the same for all graphs.

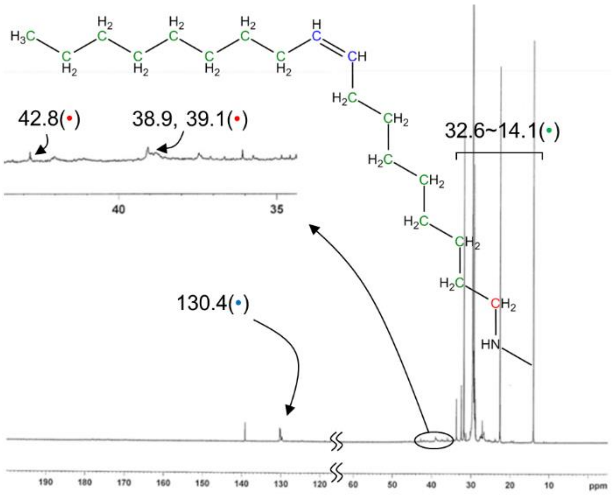

Figure S6. Nuclear magnetic resonance data of **1**. The inset shows the chemical structure of oleylamine. Color coding represents chemical bondings.

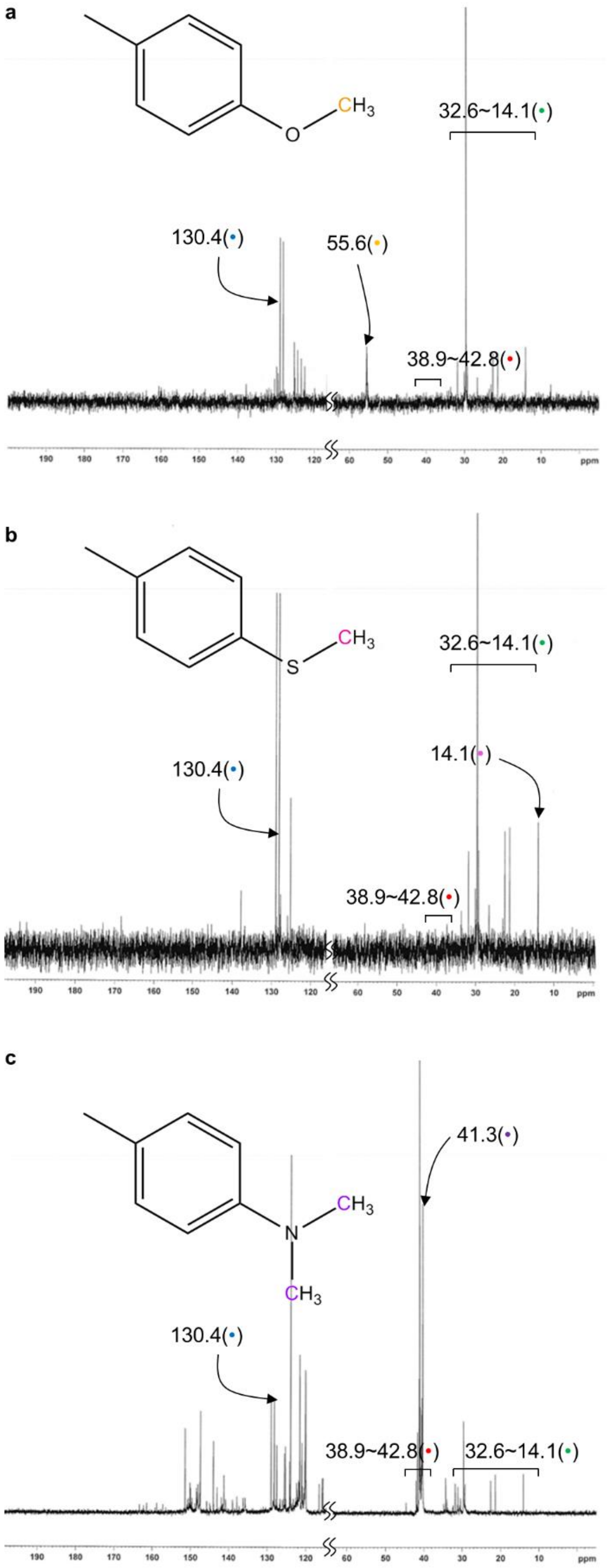

Figure S7. Nuclear magnetic resonance data of **2** (**a**), **3** (**b**) and **4** (**c**). The insets show the chemical structure of *para*-substituted anilines. Color coding represents chemical bondings.

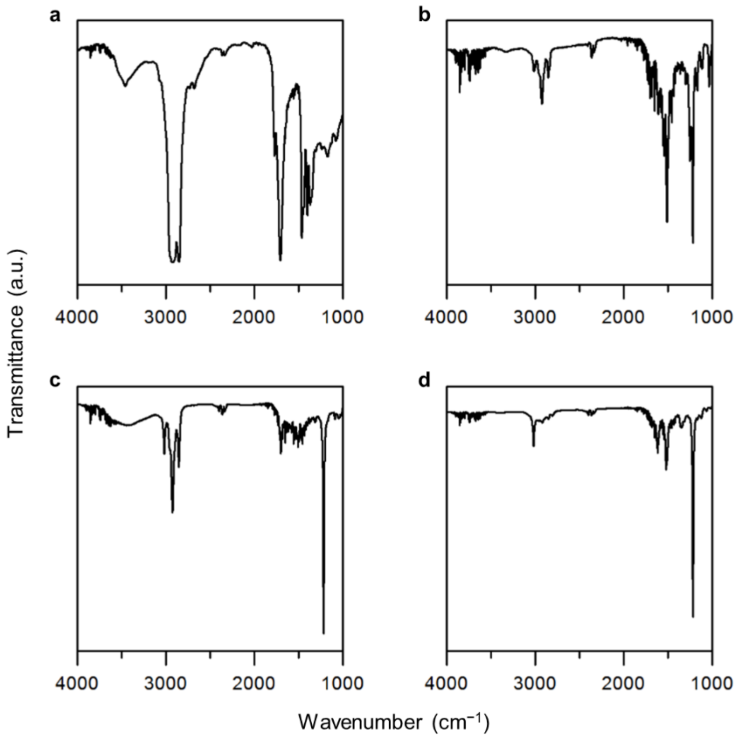

Figure S8. Infrared spectra of **1** (**a**), **2** (**b**), **3** (**c**) and **4** (**d**).

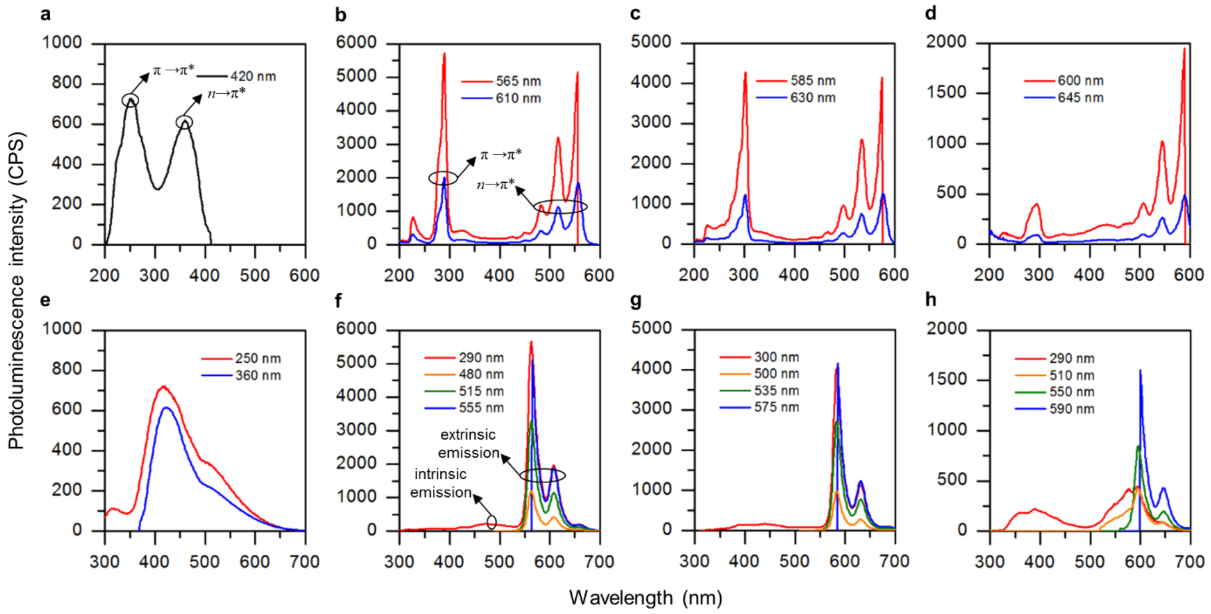

Figure S9. Photoluminescence (**a**–**d**) excitation and (**e**–**h**) emission spectra of **1** (**a**,**e**), **2** (**b**,**f**), **3** (**c**,**g**) and **4** (**d**,**h**). The legends in **a**–**d** and **e**–**h** show detection (emission) and excitation wavelengths, respectively.

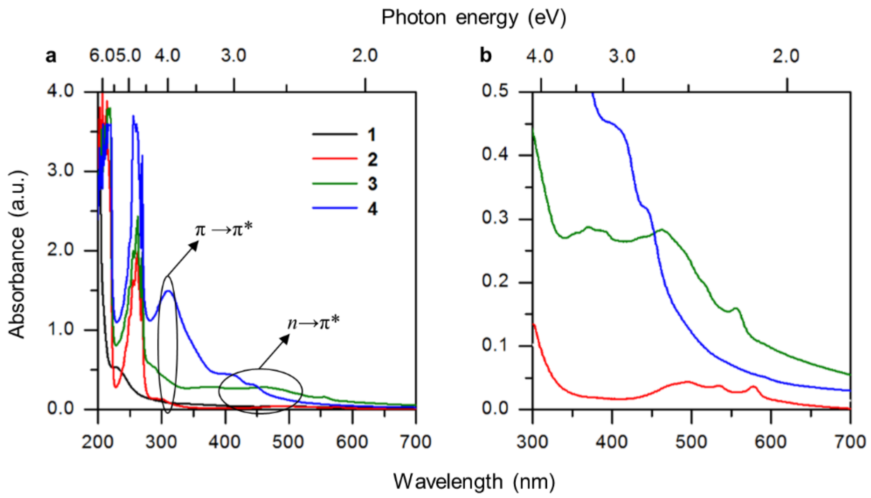

Figure S10. (**a**) Light absorption spectra of C-dots. The bands corresponding to π→π* and *n*→π* are indicated by solid circles. (**b**) The expansion of the bands corresponding to *n*→π*. Color coding is the same for all graphs.

Table S2. Energy levels of C-dots calculated by the Hückel method.

Energy level	Samples			
	**1**	**2**	**3**	**4**
LUMO+2	-	−3.071	−3.080	−1.064
LUMO+1	-	−3.086	−3.086	−3.084
LUMO[a]	−1.588	−3.600	−3.598	−3.575
HOMO[b]	−6.780	−5.924	−5.885	−5.645
HOMO−1	-	−7.583	−7.576	−7.546

[a]Lowest unoccupied molecular orbital

[b]Highest occupied molecular orbital

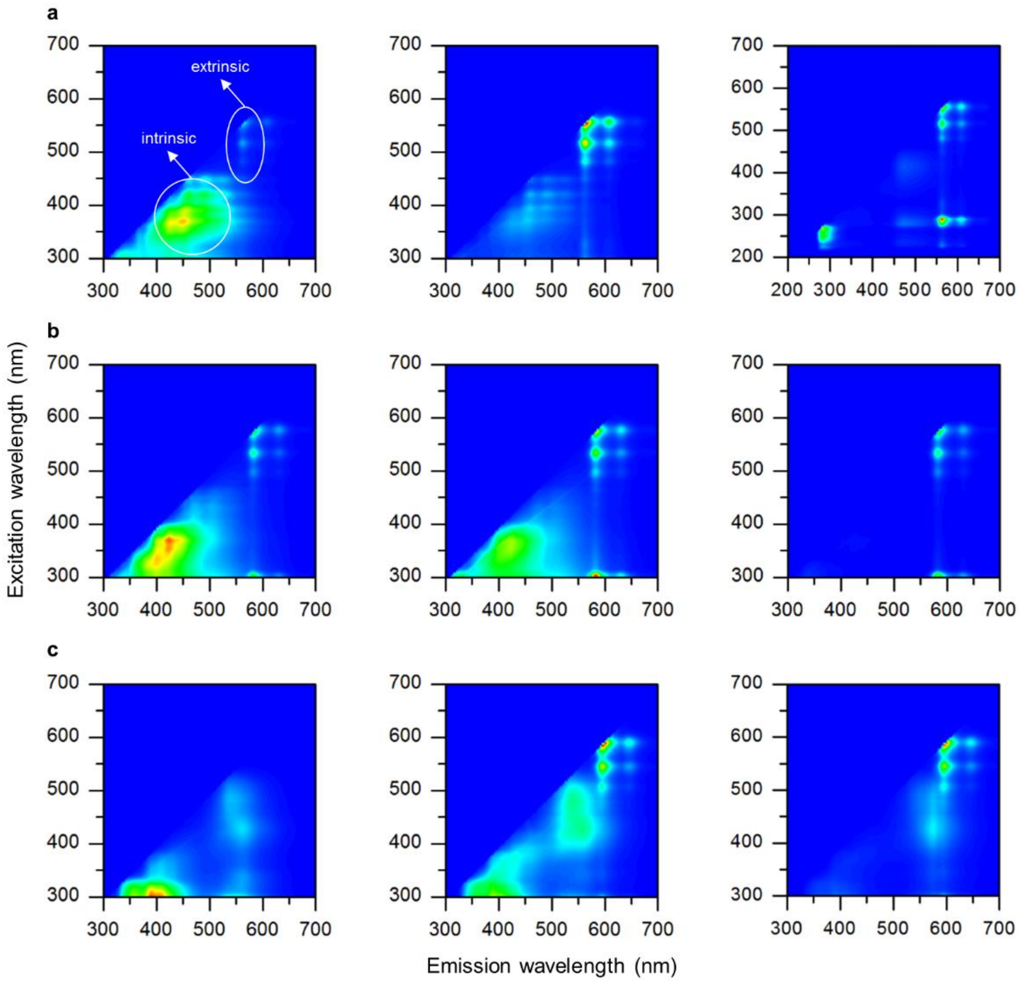

Figure S11−1. Emission maps of **2** (**a**), **3** (**b**) and **4** (**c**) as a function of the degree of surface functionalization (left column: low; middle column: medium; right column: high). The degree of surface functionalization was regulated by varying the concentration of *para*-substituted anilines. Intrinsic and extrinsic emissions were indicated by solid circles.

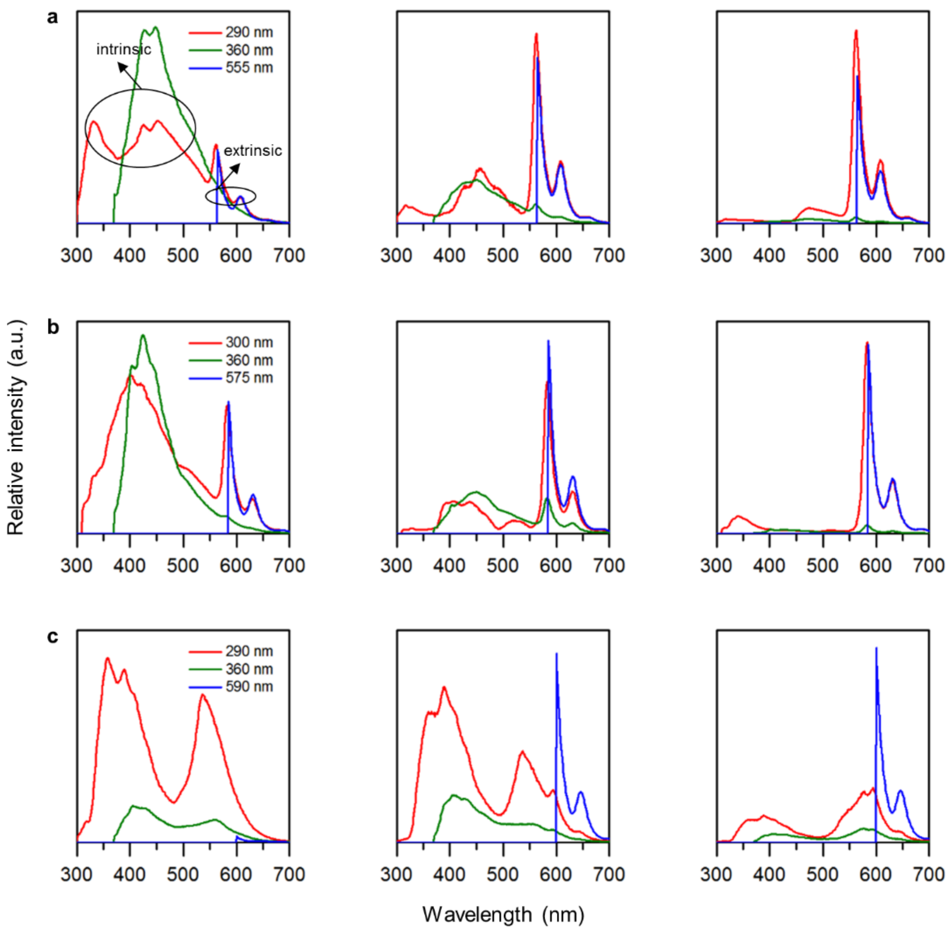

Figure S11−2. Emission spectra of **2** (**a**), **3** (**b**) and **4** (**c**) as a function of the degree of surface functionalization (left column: low; middle column: medium; right column: high). The legends show excitation wavelengths. Intrinsic and extrinsic emissions were indicated by solid circles.

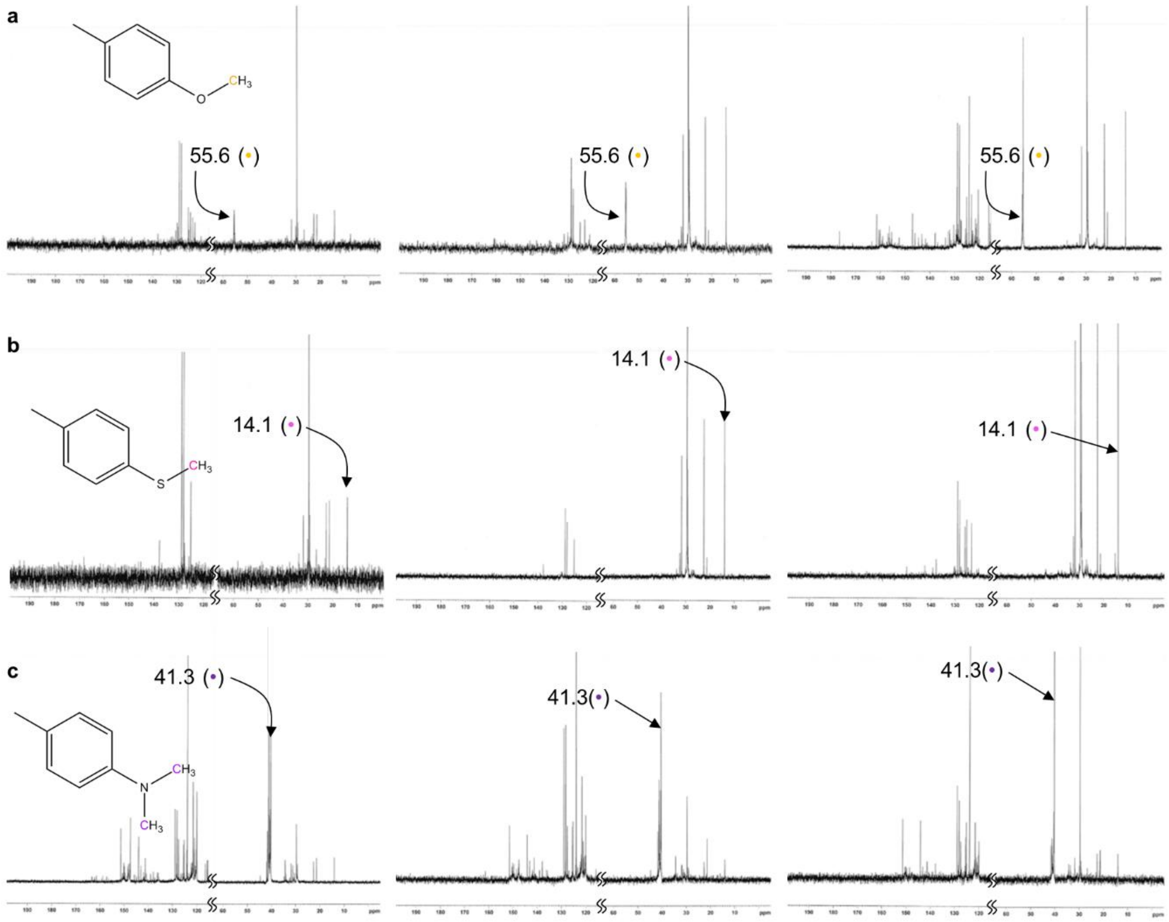

Figure S12−1. Nuclear magnetic resonance data of **2** (**a**), **3** (**b**) and **4** (**c**) as a function of the degree of surface functionalization (left column: low; middle column: medium; right column: high). The arrows indicate signals related with important chemical bondings to reflect the degree of surface functionalization.

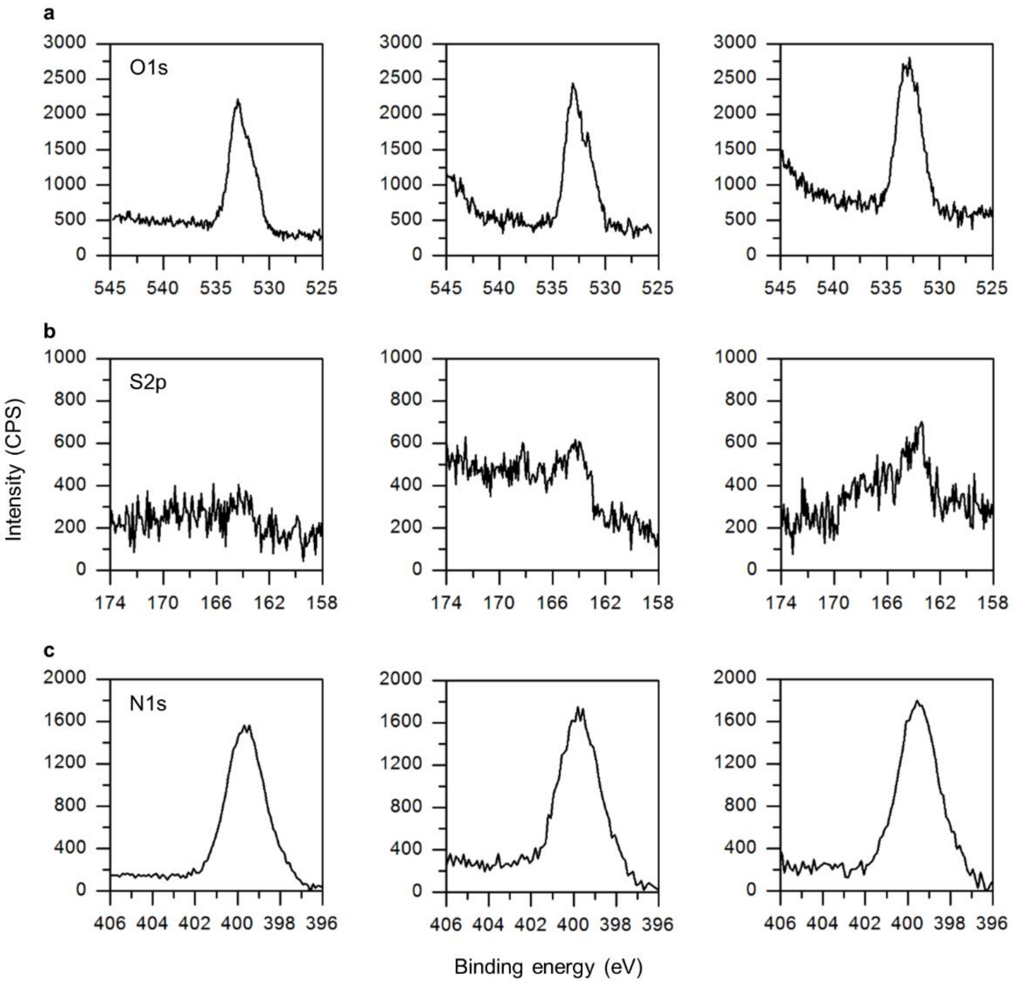

Figure S12−2. X-ray photoelectron spectra of **2** (**a**), **3** (**b**) and **4** (**c**) as a function of the degree of surface functionalization (left column: low; middle column: medium; right column: high). To determine the degree of surface functionalization, we traced the change in the number of the characteristic atoms of *para*-substituents (O, S and N for **2**, **3** and **4**, respectively).

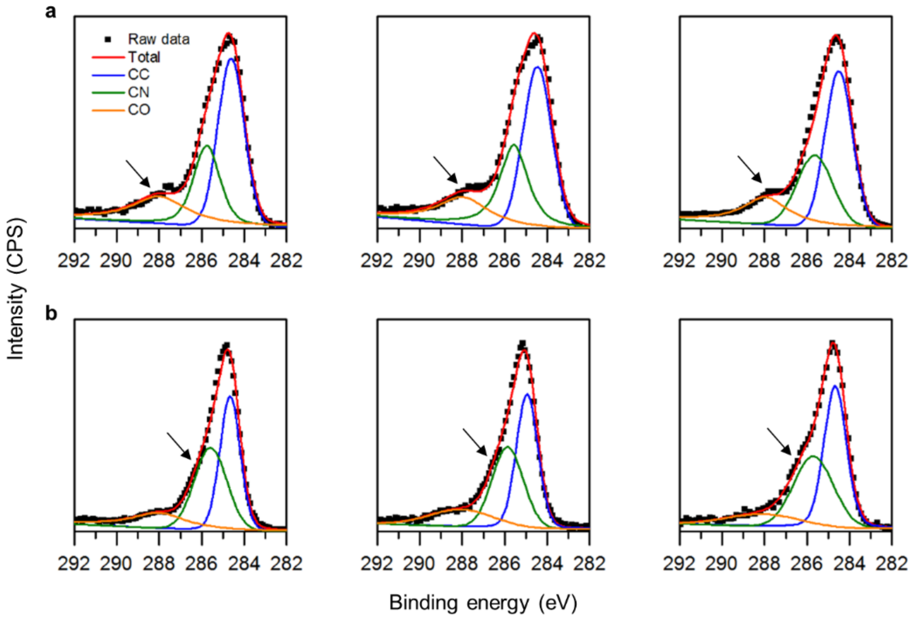

Figure S12−3. X-ray photoelectron spectra (C1s) of **2** (**a**) and **4** (**b**) as a function of the degree of surface functionalization (left column: low; middle column: medium; right column: high). The arrows indicate the increase in the number of CO and CN bondings in **2** and **4**, respectively, with the degree of surface functionalization.

Table S3. Atomic ratios of C-dots as a function of the degree of surface functionalization.

Sample	Degree	Atomic ratio (%)			
		C1s	N1s	O1s	S2p
**2**	Low	83.19	5.55	11.24	~0
	Medium	80.41	5.66	13.67	~0
	High	77.91	6.82	14.30	~0
**3**	Low	82.56	4.21	10.96	0.81
	Medium	84.68	4.58	8.60	1.06
	High	82.69	6.74	9.06	1.37
**4**	Low	75.10	12.67	11.94	~0
	Medium	73.42	12.92	11.91	~0
	High	71.66	14.54	13.56	~0

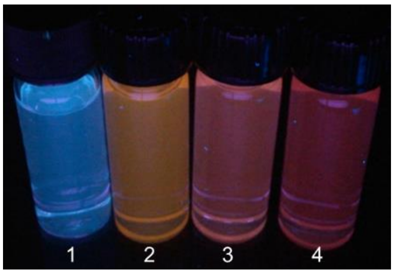

Figure S13. Photo of C-dots dissolved in hexane under 360 nm UV irradiation.

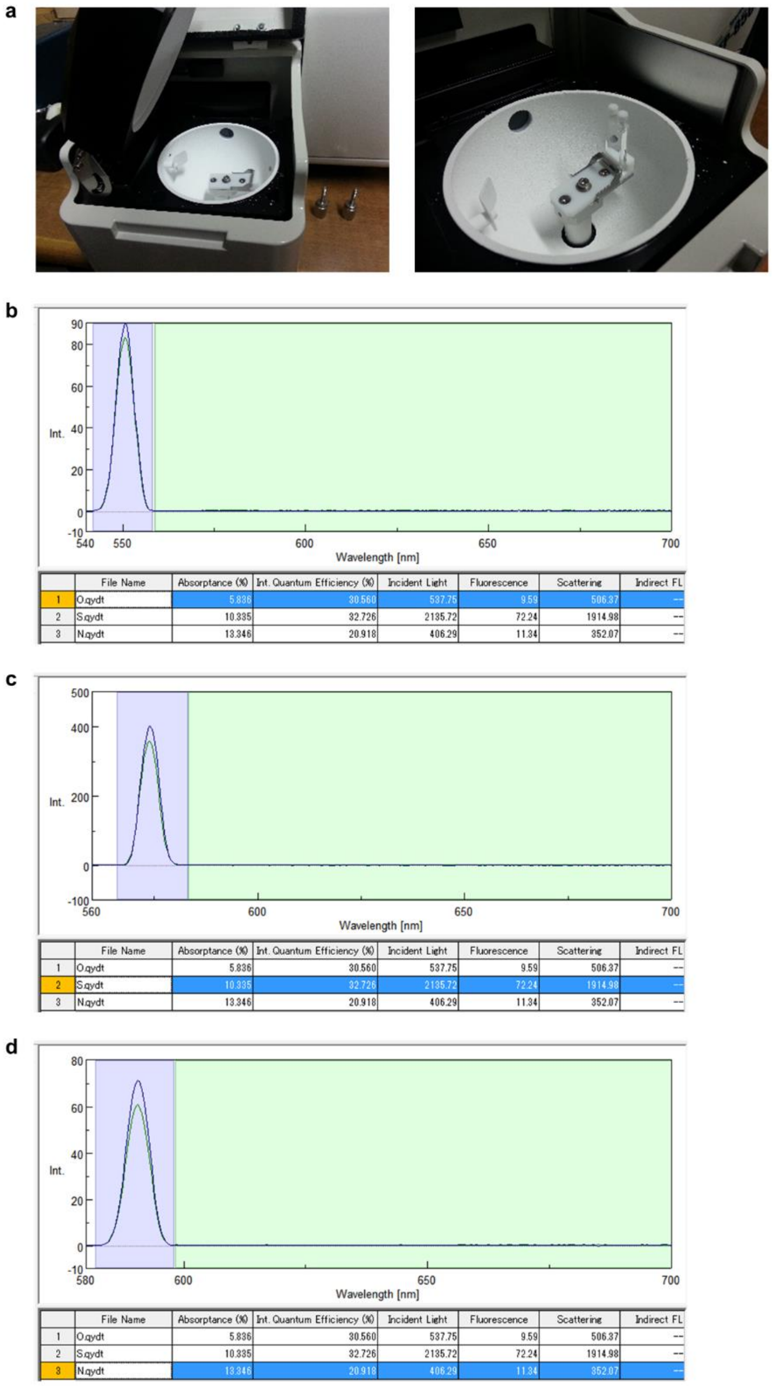

Figure S14. (**a**) Photo of integrating sphere setup. (**b−d**) Quantum yield calculation of **2** (**b**), **3** (**c**) and **4** (**d**) by using Jasco Spectra Manager II Software.

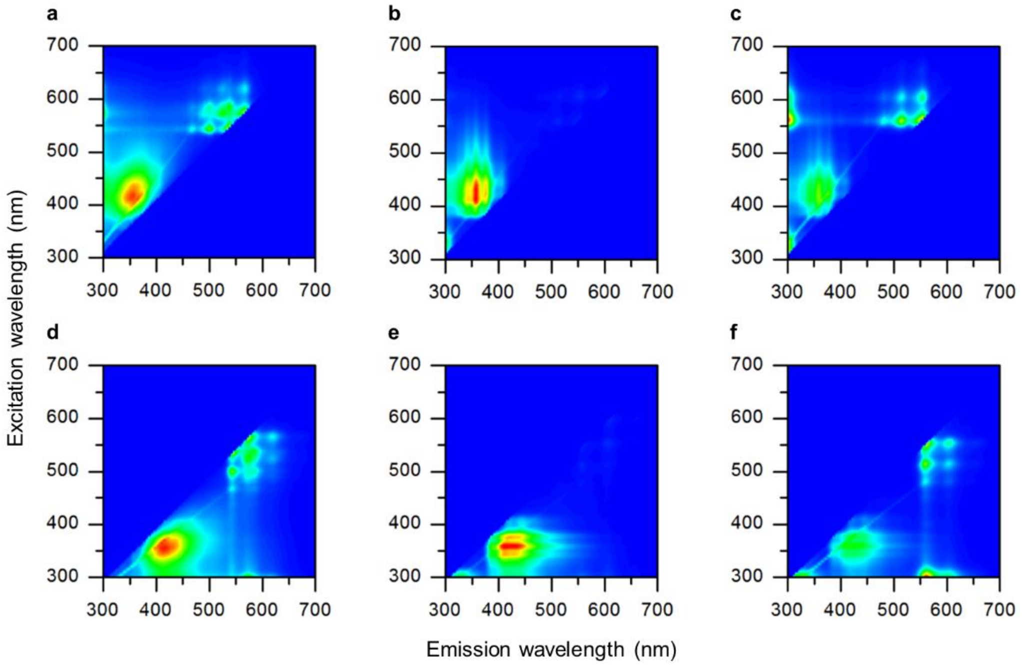

Figure S15. (**a**–**c**) Excitation and (**d**–**f**) emission maps of C-dots functionalized with 4-fluoroaniline (**a**,**d**), 4-chloroaniline (**b**,**e**) and 4-bromoaniline (**c**,**f**). The halogenated anilines were introduced under the same conditions as for **2**–**4**. The photoluminescence intensity of C-dots with the halogenated anilines was less than one-tenth of that of **2**–**4**.

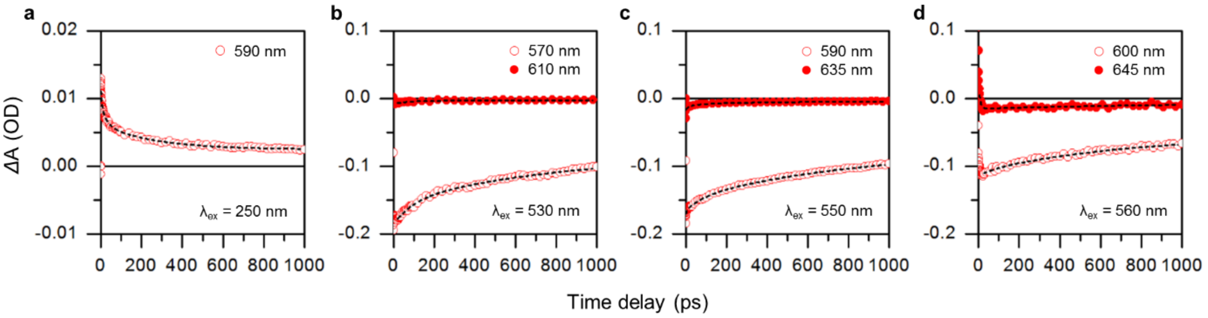

Figure S16. Time-domain TA spectra of **1** (**a**), **2** (**b**), **3** (**c**) and **4** (**d**) at different excitation wavelengths (λ_{ex}). The data were fitted to bi-exponential functions to calculate decay times (dotted lines). The legends show the wavelengths of probe light.

Table S4. Decay times of photoexcited electrons in C-dots.

Sample	λ_{probe} (nm)	τ_1 (ps)	τ_2 (ps)
**1**	590	29.27	303.30
**2**	570	93.68	1010.00
	610	83.75	882.90
**3**	590	98.36	1332.00
	635	49.12	850.90
**4**	600	93.88	950.80
	645	72.65	767.20

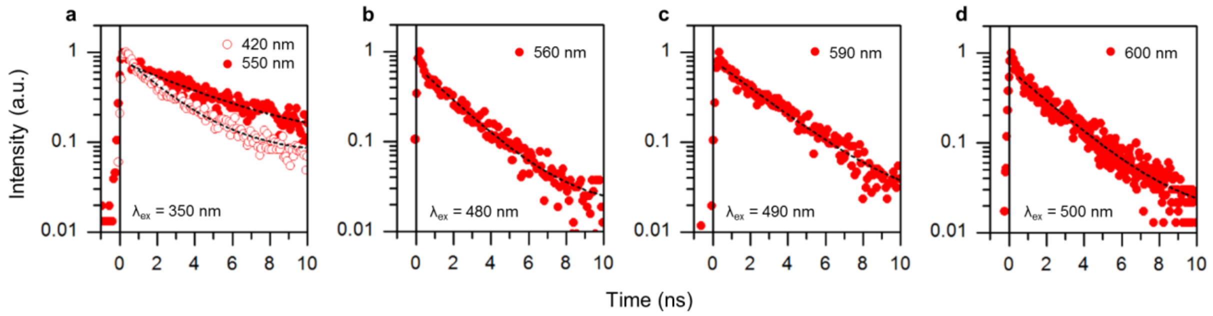

Figure S17. TRPL spectra of **1** (**a**), **2** (**b**), **3** (**c**) and **4** (**d**) at excitation wavelengths (λ_{ex}) of 350, 480, 490, and 500 nm, respectively. The data were fitted to single exponential functions to calculate photoluminescence lifetimes (dotted lines). The legends show the wavelengths of probe light.

Table S5. Photoluminescence lifetimes of C-dots.

Sample	λ_{probe} (nm)	τ (ns)
**1**	420	2.29
	550	4.29
**2**	560	2.18
**3**	590	2.73
**4**	600	2.38

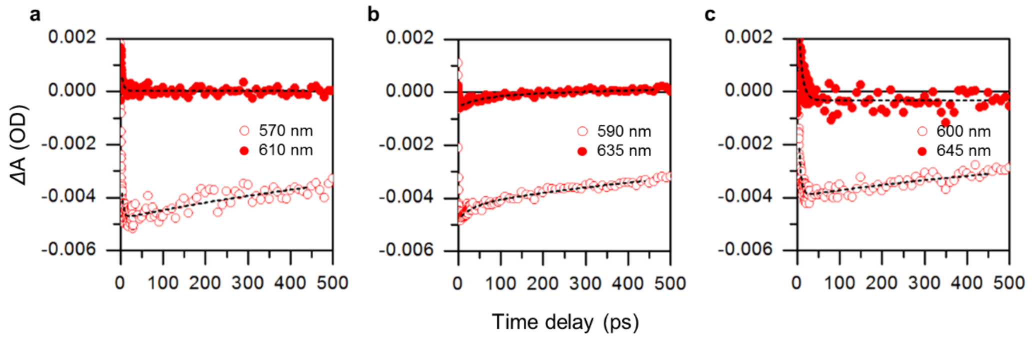

Figure S18. Time-domain TA spectra of **2** (**a**), **3** (**b**) and **4** (**c**) at the excitation wavelength of 300 nm. The data were fitted to bi-exponential functions to calculate decay times (dotted lines). The texts represent the wavelengths of probe light.

Table S6. Decay times of 300 nm photoexcited electrons in C-dots.

Sample	λ_{probe} (nm)	τ_1 (ps)	τ_2 (ps)
**2**	570	4.26	899.2
	610	5.34	-
**3**	590	3.22	824.1
	635	5.39	859.1
**4**	600	4.97	972.6
	645	8.44	-

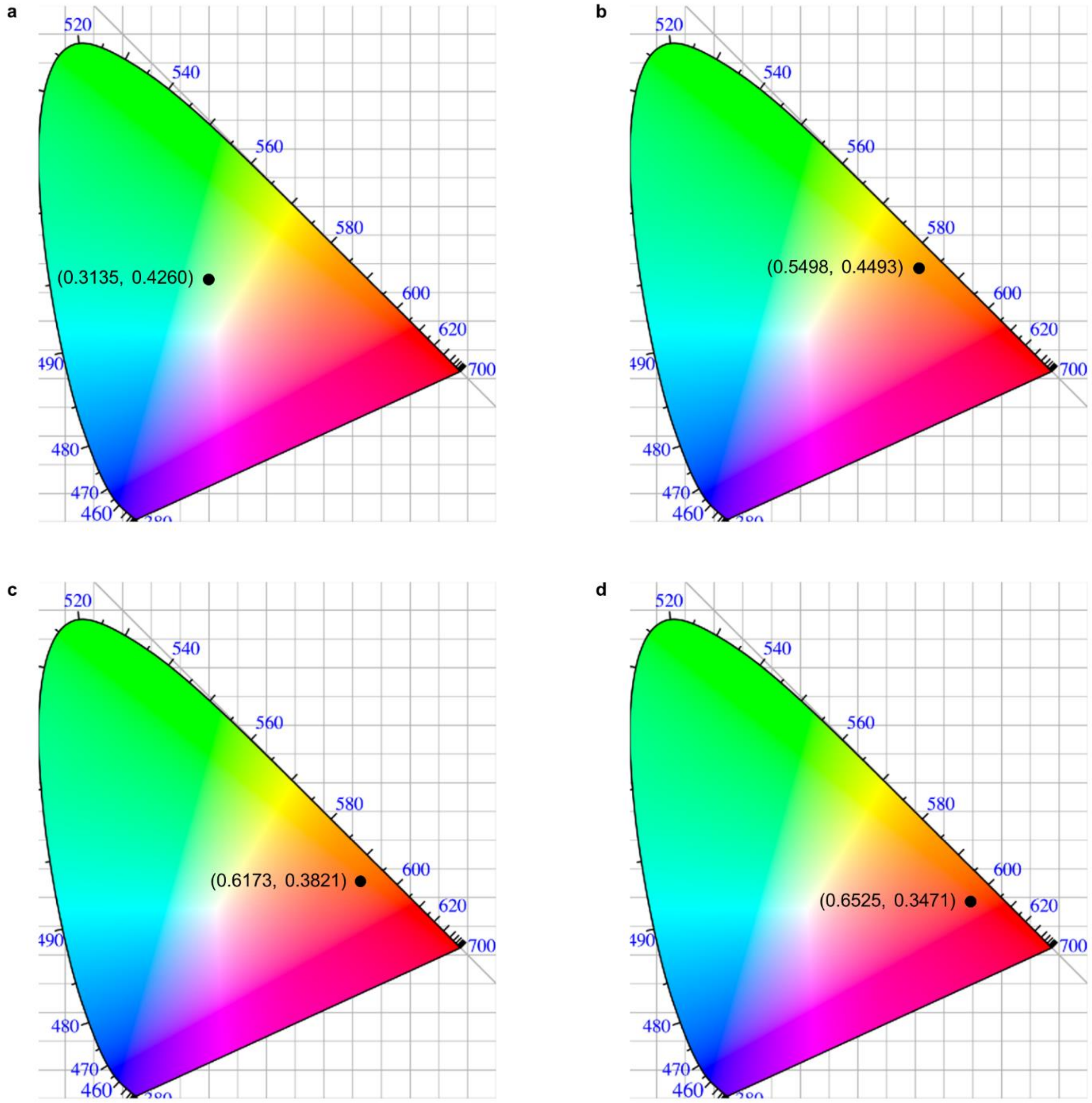

Figure S19−1. CIE chromaticity coordinates of LEDs with **f1** (**a**), **f2** (**a**), **f3** (**c**) and **f4** (**d**), calculated by using Chromaticity Coordinate Calculator (developed by Prashant Patil).

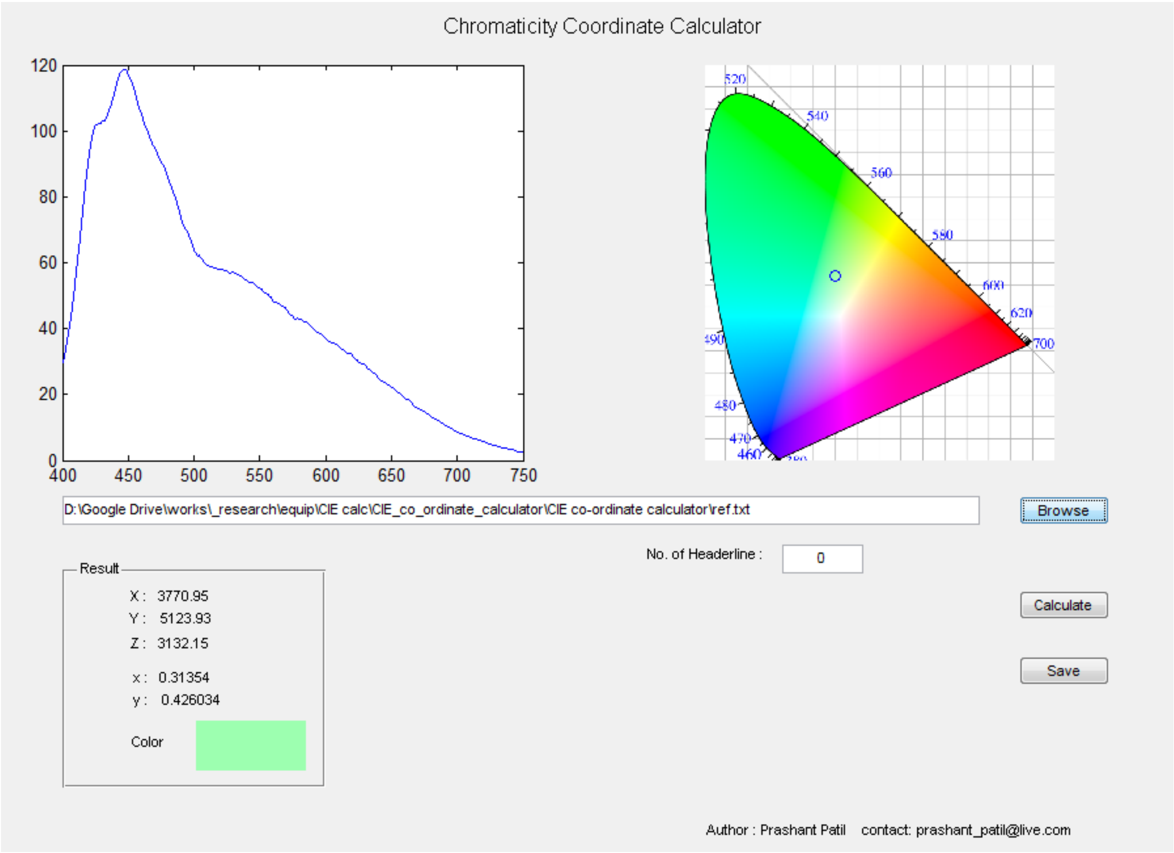

Figure S19−2 Calculation of CIE chromaticity coordinates of LEDs with **f1**.

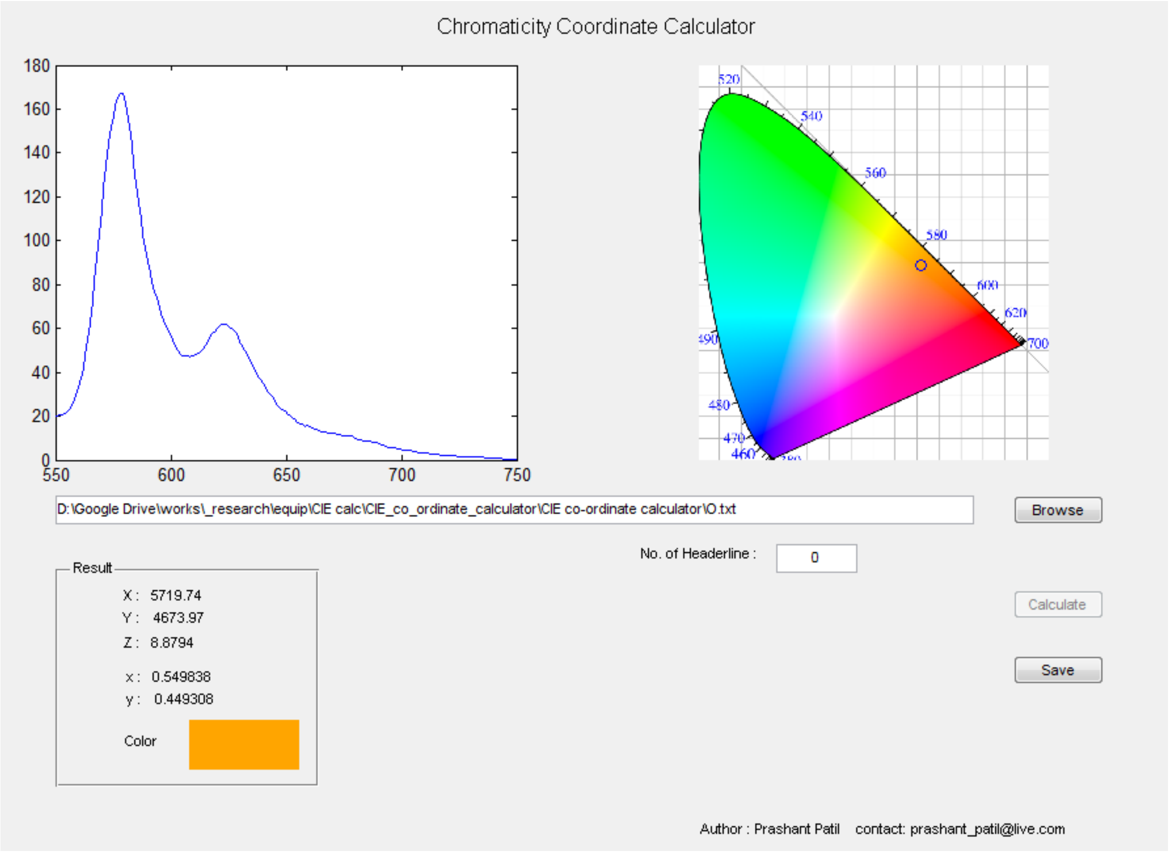

Figure S19−3 Calculation of CIE chromaticity coordinates of LEDs with **f2**.

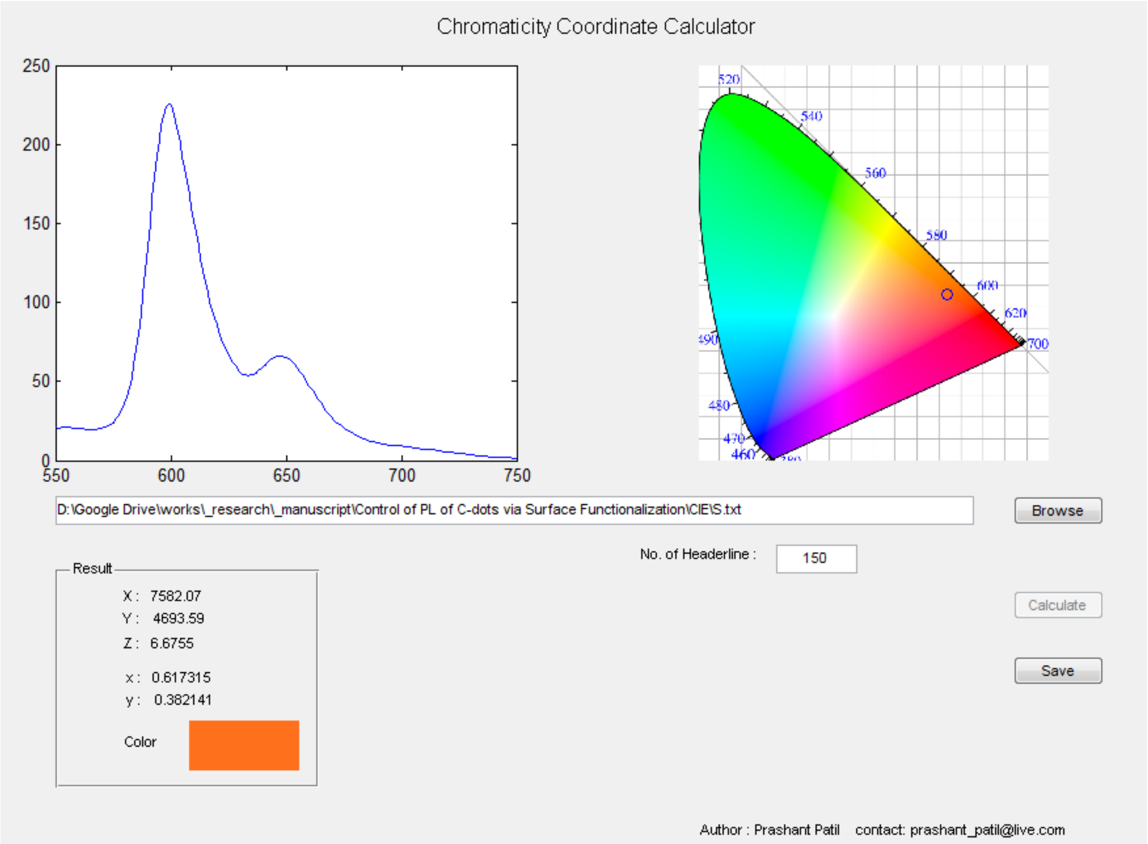

Figure S19−4 Calculation of CIE chromaticity coordinates of LEDs with **f3**.

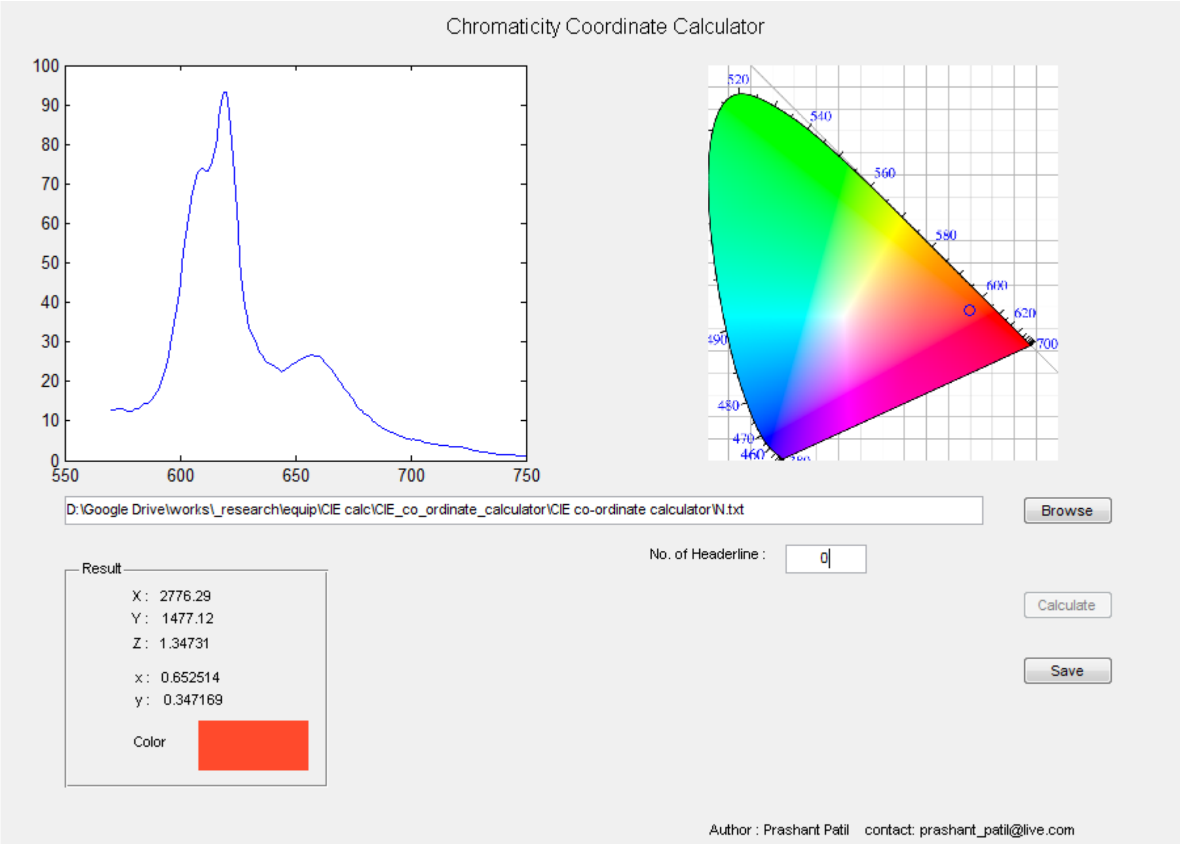

Figure S19−5. Calculation of CIE chromaticity coordinates of LEDs with **f4**.

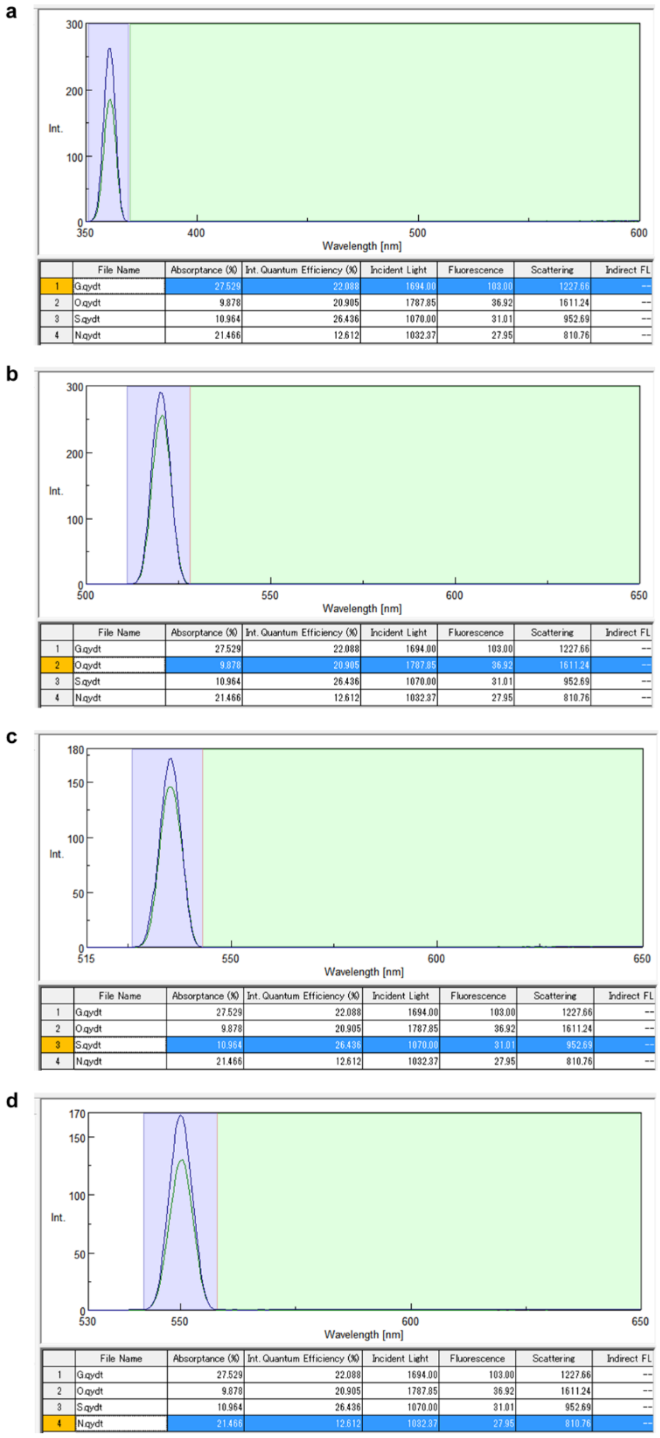

Figure S20. Quantum yield calculation of **f1** (**a**), **f2** (**b**), **f3** (**c**) and **f4** (**d**) by using Jasco Spectra Manager II Software.

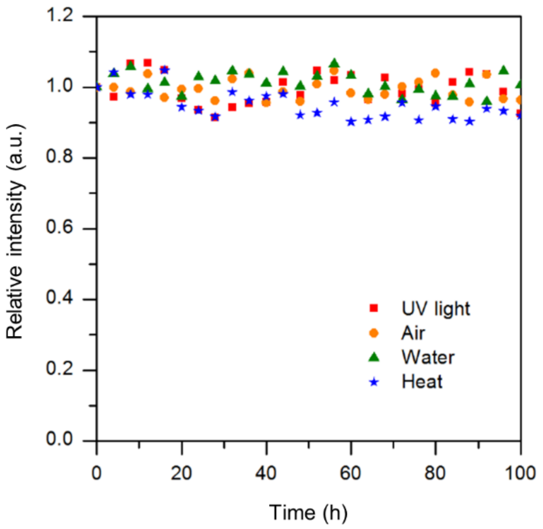

Figure S21. Long-term stability of light-converting films against UV light (360 nm), air, water and heat (80 ℃). 360 nm UV lamps and digital hot plates were employed for exposing UV light and heat, respectively. For testing air-stability, the films were placed on silica gel under ambient conditions. For testing water-stability, the films were immersed in tap water at room temperature. In the experiments, the photoluminescence intensity of our films was recorded every 4 h on a Jasco FP-8500 fluorometer.

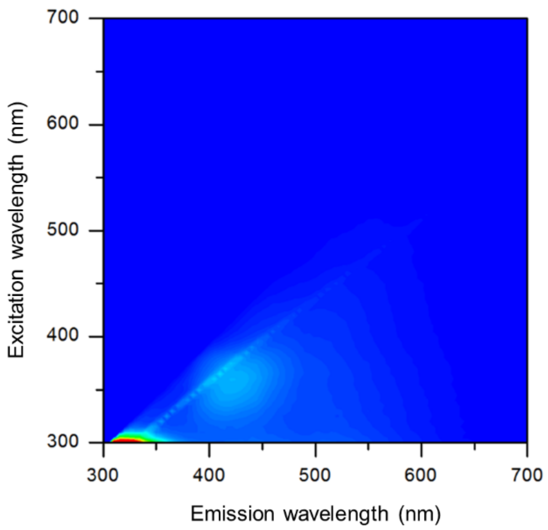

Figure S22. Emission map of C-dots functionalized with aniline (no substituent).

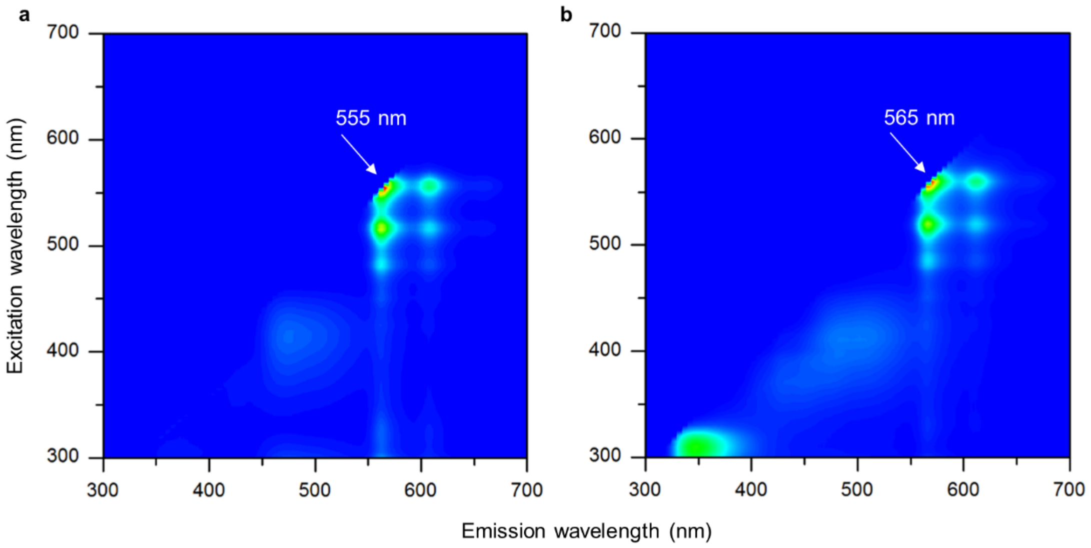

Figure S23. Emission maps of C-dots functionalized with 4-methoxyaniline (**a**) and 4-(octyloxy)aniline (**b**).

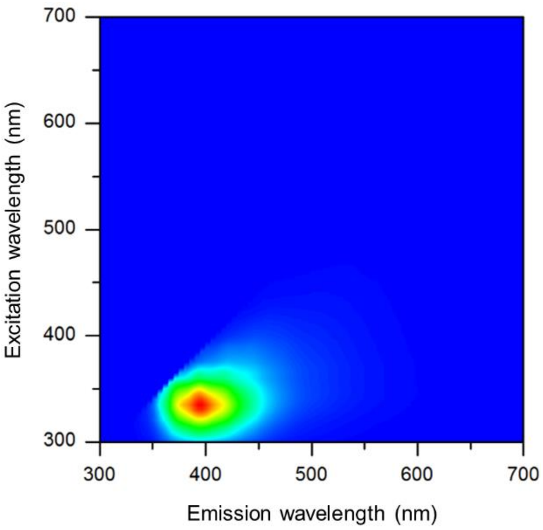

Figure S24. Emission map of C-dots functionalized with 4-methoxybenzylamine.

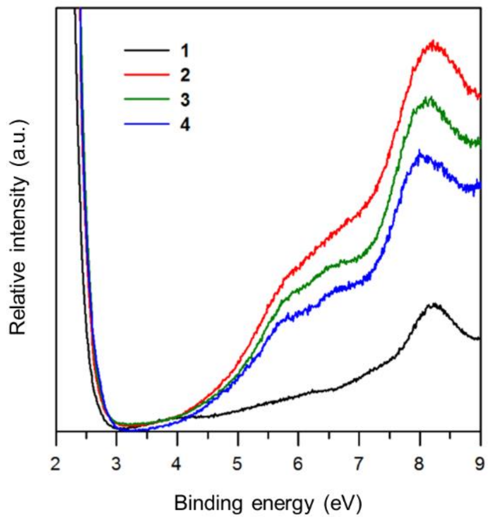

Figure S25. Electron energy loss spectroscopy data of C-dots deposited on gold-coated silicon substrates.

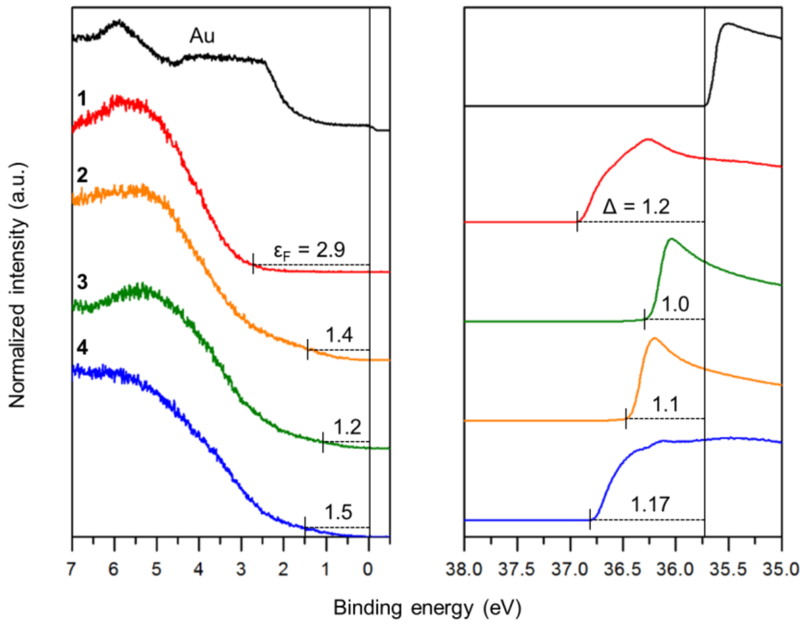

Figure S26. Ultraviolet photoelectron spectroscopy data of C-dots deposited on gold-coated silicon substrates.

Ishii and coworkers have suggested a mathematical method to extract the highest occupied molecular orbital (HOMO) level of organic semiconductor deposited on a metal film (typically Au) from ultraviolet photoelectron spectroscopy. The HOMO level (E_{HOMO}) can be calculated by using the following equation:

$$E_{HOMO} = \varepsilon_F + \Phi_m - \Delta$$

, where ε_F is the difference between the Fermi edges of metal and organic semiconductor, Φ_m is the work function of metal (5.1 eV for Au), and Δ is the vacuum shift (the difference between the secondary cutoffs of metal and organic semiconductor). We note that ε_F and Δ are due to higher electron-binding energy of organic semiconductor relative to metal and the presence of a dipole moment at the interface, respectively. The extracted HOMO levels of **1**, **2**, **3**, and **4** were then 6.8, 5.5, 5.2, and 5.4 eV, respectively.

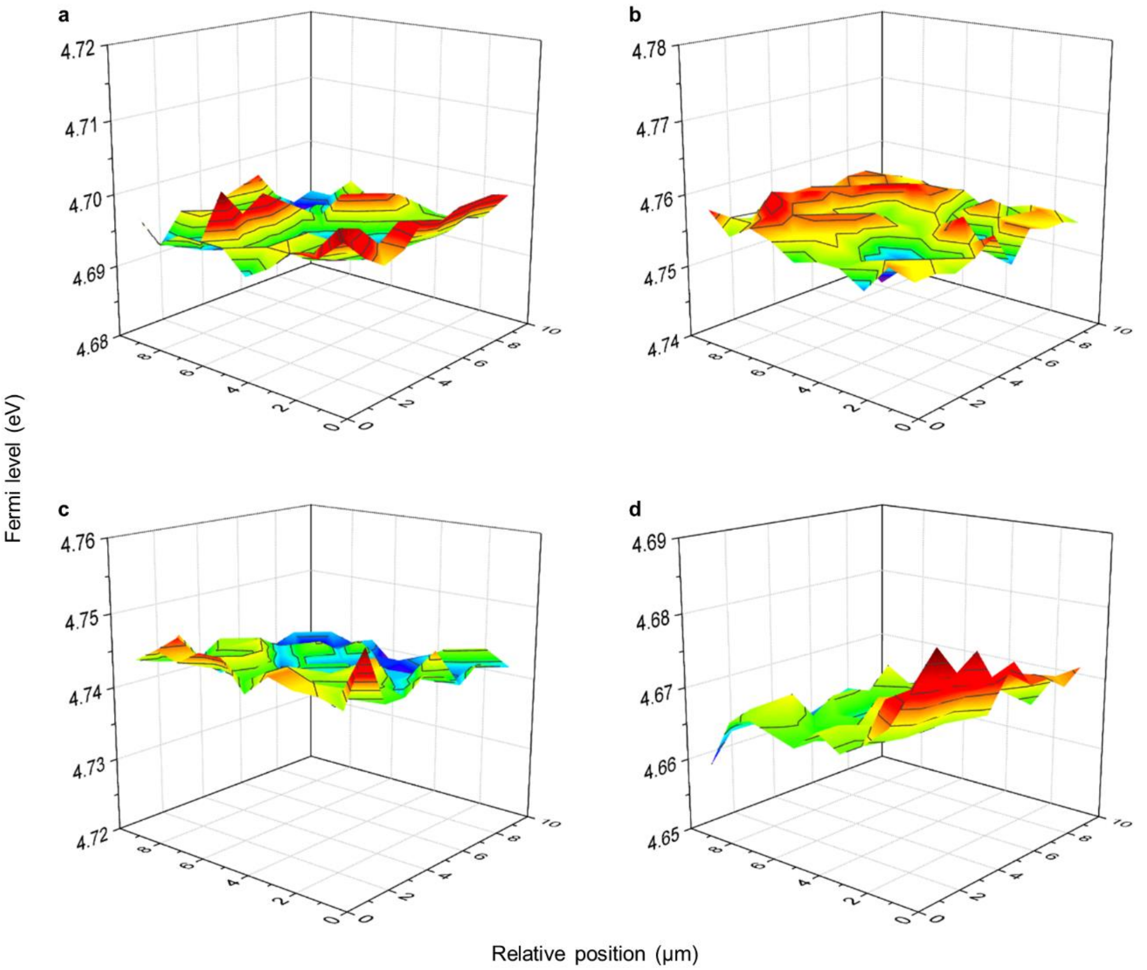

Figure S27. Kelvin probe analysis data of C-dots deposited on gold-coated silicon substrates.

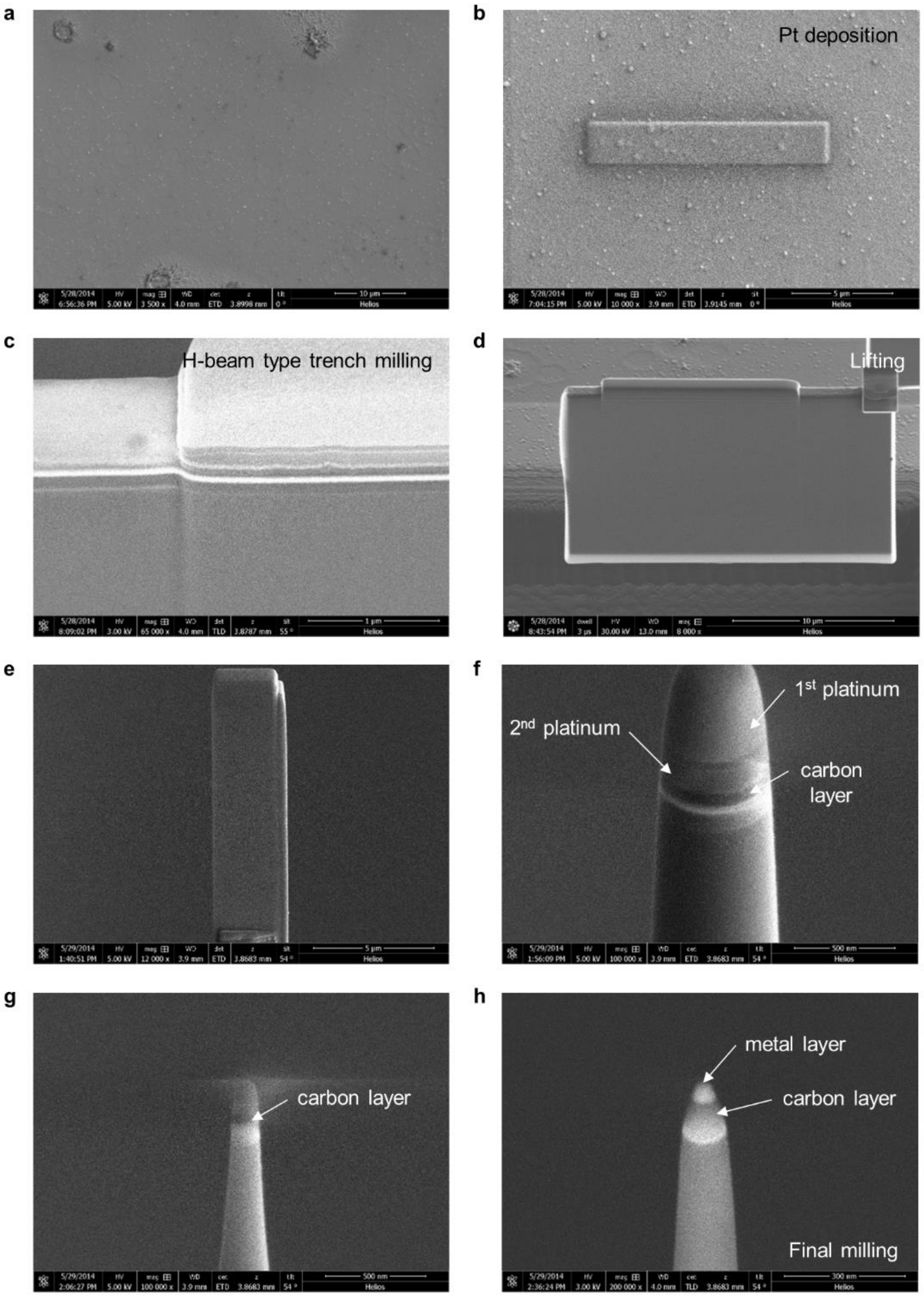

Figure S28. Preparation of atom probe specimens: scanning electron microscopy image of C-dots deposited on platinum substrates (**a**); platinum deposition (**b**); trench milling (**c**); lifting (**d**); side view of a lifted sample (**e**); step-by-step milling (**f**−**h**).
